# Three-Dimensional Cathodes for Electrochemical Reduction of CO_2_: From Macro- to Nano-Engineering

**DOI:** 10.3390/nano10091884

**Published:** 2020-09-20

**Authors:** Shiqiang (Rob) Hui, Nima Shaigan, Vladimir Neburchilov, Lei Zhang, Kourosh Malek, Michael Eikerling, Phil De Luna

**Affiliations:** 1 Energy, Mining and Environment, National Research Council Canada, Vancouver, BC V6T 1W5, Canada; Nima.Shaigan@nrc-cnrc.gc.ca (N.S.); Vladimir.Neburchilov@nrc-cnrc.gc.ca (V.N.); Lei.Zhang@nrc-cnrc.gc.ca (L.Z.); Kourosh.Malek@nrc-cnrc.gc.ca (K.M.); Phil.DeLuna@nrc-cnrc.gc.ca (P.D.L.); 2 Institute of Energy and Climate Research, IEK-13: Modelling and Simulation of Energy Materials, Forschungszentrum Jülich, 52425 Jülich, Germany; m.eikerling@fz-juelich.de

**Keywords:** three dimensional electrodes, cathodes, electrochemical reduction of CO_2_, electro-catalysis, nanomaterials, modeling, CO_2_ use

## Abstract

Rising anthropogenic CO_2_ emissions and their climate warming effects have triggered a global response in research and development to reduce the emissions of this harmful greenhouse gas. The use of CO_2_ as a feedstock for the production of value-added fuels and chemicals is a promising pathway for development of renewable energy storage and reduction of carbon emissions. Electrochemical CO_2_ conversion offers a promising route for value-added products. Considerable challenges still remain, limiting this technology for industrial deployment. This work reviews the latest developments in experimental and modeling studies of three-dimensional cathodes towards high-performance electrochemical reduction of CO_2_. The fabrication–microstructure–performance relationships of electrodes are examined from the macro- to nanoscale. Furthermore, future challenges, perspectives and recommendations for high-performance cathodes are also presented.

## 1. Introduction

The continued exploration and development of renewable technology is critical for addressing the worldwide problem of anthropogenic CO_2_ emissions. CO_2_ can be converted into valuable chemicals and fuels through diverse routes, including biochemical, electrochemical, photochemical, radiochemical, and thermochemical reactions [1,2,3,4,5,6]. Among these CO_2_ conversion technologies, electrochemical reduction of CO_2_ coupled with renewably generated electricity from wind, solar, or hydroelectricity provides an attractive approach for carbon-neutral production of fuels and chemicals [7,8,9,10]. Significant technical progress has been made in recent years, and preliminary technoeconomic analysis of CO_2_ electrochemical reduction has demonstrated the commercial feasibility of the technology [11,12,13,14,15]. Electrochemical reduction of CO_2_ generates various gas and liquid products depending on the number of electrons and protons transferred. Some of the major products are summarized in Table 1, along with electrochemical reactions and their associated equilibrium potentials [16].

**Figure 1:**
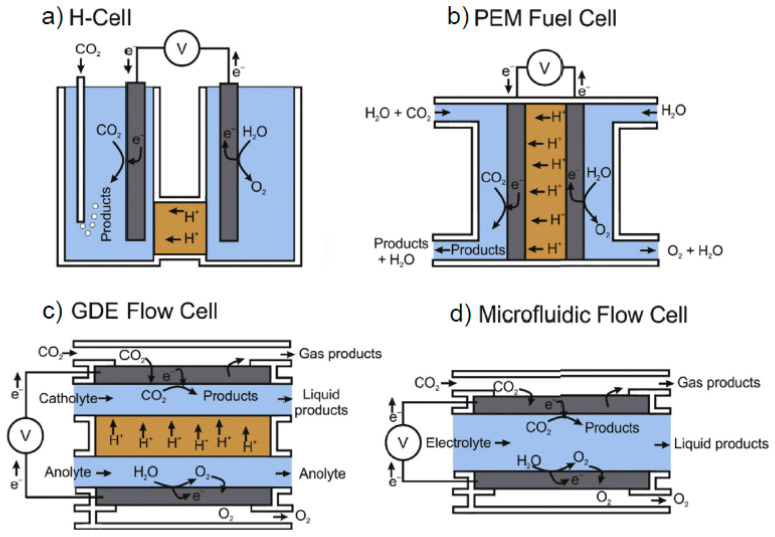
Illustration of reactor designs commonly used for the electrochemical reduction of CO_2_ [19]. Reproduced from open access. Elsevier, 2020.

To date, electrochemical reduction of CO_2_ has been demonstrated by systems operating under ambient conditions (H-cell, flow cells, membrane-electrode-assembly (MEA) cells) or under high temperatures such as solid oxide electrolysis cells (SOECs) [17,18,19,20]. Figure 1 illustrates configurations of different reactor designs commonly used for the electrochemical reduction of CO_2_ [19]. CO_2_ can be flowed into the cathodic compartment, either via dissolution in electrolyte or as humidified gas in a MEA cell. A gas diffusion electrode flow cell has the catholyte and anolyte separated via an ion exchange membrane, while a microfluidic flow cell only has a single electrolyte flowing between anode and cathode. The performance of electrochemical CO_2_ reduction is commonly studied by the following parameters [19]:

(1) Overpotential, the difference between the thermodynamic and actual electrode reduction voltages. Overpotential is a measure of the energy efficiencies of the system.
(2) Faradaic efficiency (*FE*), calculated by ε_Faraday_ = αnF/Q, where α is the number of electrons transferred, n is the number of moles for a given product, F is Faraday’s constant of 96,485 C mol^−1^, and Q is all the charge passed throughout the electrolysis process. *FE* describes the product selectivity in the reduction reaction, which is closely related to the reduction mechanism. Different reaction pathways are strongly affected by experimental conditions.
(3) Current density (CD), obtained by dividing the total current by the surface area of the working electrode. CD reflects the rate of CO_2_ reaction.
(4) Stability, degradation rate of CD over the period of system operation.
(5) Tafel slope, derived from the plot of overpotential against the logarithm of partial CD, is an indicator for the reaction pathway and the rate-determining step.

All of these parameters are influenced by materials and surface properties of catalysts, architectures of electrodes, electrolytes, and operating conditions such as temperature and pressure.

Cathodes in the system play a crucial role since CO_2_ reduction reactions (CO_2_RRs) occur on the surface of the cathode electrocatalyst as follows:

$$x{\mathrm{CO}}_{2}+nH^{+}+ne^{-}\overset{\mathrm{Catalysts}}{\to }Products+yH_{2}O$$

CO_2_RR occurs only on the surfaces of a catalytic cathode where the triple phase of CO_2_, H^+^, and e^−^ may coexist. Therefore, it is critical to achieve high CD by controlling the wetting property of the cathode for high CO_2_ (and H+ in aqueous electrolyte) transfer, boosting high electronic conductivity, and enabling large surface areas by nanoengineering. The following are prerequisite properties for effective cathodes for electrochemical CO_2_ reduction:

(1) Effective electrocatalysts to provide a low overpotential and high *FE* for the desired reactions;
(2) A high electrical conductivity to minimize ohmic losses and a uniform current distribution (a mixed ionic and electronic conduction (MIEC) architecture benefits extension of reaction sites);
(3) Porous architecture engineered from the macro- to nano-level to facilitate higher charge and mass transfer;
(4) Mixed hydrophobic and hydrophilic properties for gas diffusion electrodes to avoid flooding while providing adsorptions;
(5) Good mechanical and chemical stability to ensure an adequate lifetime;
(6) Moderate costs.

With these parameters as measurement targets toward commercialization, stable long-term operation over 20,000 h at substantial CDs greater than 200 mA cm^−2^ with efficiencies over 70% remains a challenge for the electrochemical reduction of CO_2_ [21,22]. To date, the longest continuous operation of electrochemical reduction of CO_2_ was demonstrated at an applied CD of 200 mA cm^−2^ for over 1000 h and at 50 mA cm^−2^ for 4380 h using an MEA-based electrolyzer in 2017 [23]. In addition, the total surface area of the cathode is only 6.25 cm^2^, which is far too small for commercial applications. It has been challenging to achieve high production rates (CDs), high selectivity (*FE* for the desired products), high energy efficiencies (low overpotentials), high single pass conversion efficiencies (ratio of converted/unconverted CO_2_ in a single pass) and long-term stable performance simultaneously. Performance improvements mainly depend on breakthroughs on novel catalytic materials and cathode architectures, suitable electrolyte materials, and system design (separation steps) [24]. Challenges and solutions in terms of overpotential, selectivity, CD, and stability are briefly summarized in the following paragraphs.

The stable and chemically inert linear molecular structure of CO_2_ leads to a high thermodynamic activation energy of reduction, limited concentration of reactants and multi-proton-coupled electron transfer steps kinetically [25]. A reduction reaction with a more positive *E*° in Table 1 is thermodynamically more favorable, according to the relationship ΔG = −*nFE*°, where n is the number of electrons transferred during the redox reaction and F is the Faraday constant. Homogeneous and heterogeneous catalysts have been applied to the electrochemical reduction of CO_2_ [26,27]. Most homogeneous catalysts suffer from significant drawbacks, such as high cost, toxicity, poor stability, and complex post-separation, limiting practical application. However, high selectivity can be achieved in homogeneous catalysis [28,29]. Therefore, development has been mainly focused on heterogeneous catalysts such as metals, metal oxides and sulfides, carbon-based materials, and metal-organic frameworks. Recently, as an emerging frontier in the catalysis community, single-atom catalysts (SACs) with supported metal atoms anchored by covalent coordination have exhibited enhanced activity due to tuning the metal electronic structure and high specific surface area [30,31]. Similar to molecular catalysts, SACs possess a well-defined and specific atomic structure that can offer high selectivity towards the adsorption/desorption of certain intermediates during the electrochemical reduction of CO_2_. SACs build a conceptual bridge between heterogeneous and homogeneous catalysis and offer an opportunity to design and understand heterogeneous catalysis from a molecular perspective [32,33]. An ideal catalyst will minimize the activation barrier for CO_2_ reduction in relation to proton reduction, drive CO_2_ reduction selectively at a low overpotential (i.e., high energy efficiency), and provide ample reaction sites for satisfactory reaction rates (i.e., high turnover number). Since most mass transfer processes occur on the surface, design of catalysts with nanoengineered surfaces and macro scale three dimensional porous structures is necessary for the effective performance of catalysts. There are a large number of surface morphology factors impacting catalytic activity such as composition, morphology, intermediate binding energy, packing, kinetic supply of reactants, desorption of products, and adsorbate–adsorbate interactions [34,35,36,37,38,39,40,41,42,43,44,45]. The adsorption energies of reaction intermediates follow linear scaling relationships that limit catalytic efficiency in the multi-proton-electron transfer reduction of CO_2_ [46,47]. To improve catalytic activity, different strategies have been suggested to break these linear scaling relations, including reducing coordination numbers, doping with p-block elements, introducing oxophilic sites, and coating the catalyst surface with active ligands [47]. The catalytic activity is also sensitive to the changes in local pH, electrolytes, and cations [48,49,50,51,52,53,54,55,56,57,58]. The ability of a catalyst to reduce the energy barrier for CO_2_ reduction is a result of combined intrinsic material and extrinsic environmental effects, beyond just the surface properties of the catalyst [21].

The majority of literature has reported overpotential as a total contribution from the system when studying performance of catalysts. However, it should be noted that overpotential is a sum of the activation overpotential from the performance of a catalyst, the concentration overpotential, and resistance overpotential [59,60,61]. The movement of ions in the electrochemical cell is controlled by migration, convection, and diffusion. Ions migrate toward the electrolyte-electrode interface and form a double layer of concentrated ions which acts as a concentration barrier. The extra energy required for the movement of the ions to and through this double layer is the concentration overpotential. Resistance overpotential is generally regarded as the potential drop of ohmic loss from cathode. Since additional energy must be supplied to activate the ions discharged at the required rate to promote flow of current, CO_2_ reduction with two protons transfer processes for CO and formate is typically found to have lower overpotentials compared to other hydrocarbons and oxygenates products [62]. Therefore, reduction of overpotential should solve for improvement of catalyst performance, optimization of the electrolyte-electrode interface, and strategies to enhance the conductivities of the cathode and electrolyte.

CO_2_ reductions suffer from competitive proton reduction and the generation of multiple products in the same range of applied potentials. The main selectivity issue is competitive water reduction to produce H_2_, i.e., hydrogen evolution reaction (HER). One effective approach to restrict HER is using strong alkaline electrolyte; another is to engineer an electrocatalyst to not favor HER [17]. Catalysts do not just reduce the energy barrier, but may also promote a completely different reaction pathway typically with multiple energy barriers that must be overcome and then lead to the change of product selectivity of CO_2_ reduction. In recent years, there have been significant efforts to enhance the selectivity and reactivity of electrocatalysts to engineer better catalytic performance and increased stability for the electrochemical conversion of CO_2_ [63,64,65,66,67,68,69]. Another strategy to improve the selectivity and reactivity is modulation of electrocatalyst morphologies to increase active sites, such as preferential faceting, grain boundaries, and tuning pore shape and size [35,36,39,47,70,71,72,73,74,75,76,77,78,79,80,81,82,83,84,85]. Most recently, it has been demonstrated that C_2+_ selectivity could be enhanced by tuning CO_2_ mass transport via the catalyst layer structure, feed concentration, and flow rate of CO_2_ in gas-diffusion electrode electrolyzers. Modulation of local CO_2_ concentration enabled an optimized faradaic efficiency toward C_2+_ products of up to 75.5% at 300 mA cm^−2^ in 1.0 M KHCO_3_ [86].

CDs are controlled by the mass and charge transfer of reactants. At high CD, both charge transfer and mass transfer play roles in the reaction rate while electron transfer prevails at low CD [87]. The first strategy to achieve high CD is to enhance the solubility of CO_2_ in water. One of the challenges to achieve desired high CD is the limited solubility of CO_2_ in water at acidic or neutral pH values (~0.034 M at 25°), particularly when aqueous electrolytes are used. The low CO_2_ concentrations limit mass transport and significantly hinder CO_2_ conversion rates. Different approaches have been used to enhance CO_2_ dissolution, such as the use of nonaqueous electrolytes, flow electrolyzers, and high-temperature and/or high-pressure working conditions [88,89,90,91]. High temperature and high pressure also influence the thermodynamics of the electrochemical reduction of CO_2_ and therefore enhance the performance of electrocatalysts. The second strategy to achieve high CD is to provide gaseous CO_2_ directly to reactions sites together with dissolved CO_2_ in moisture. The CD of CO_2_RR is controlled by mass transport of CO_2_ with charge transfer of H^+^ and e^-^ at these three-phase boundaries on the surfaces of cathode. Participation of both gaseous and dissolved CO_2_ in the reactions overcomes the intrinsic solubility challenges of CO_2_ to increase CD [92,93,94,95,96,97]. The third strategy is to design the architecture of the cathode to enhance the charge transfer and mass transfer, promoting high CD and long-term stability. Through architecture design, catalysts are expected to simultaneously exhibit reduction of activation overpotential, product selectivity, long-term stability and cost-effectiveness, enabling the commercial implementation of electrochemical reduction of CO_2_. Additionally, the surface and architecture of the cathode catalyst significantly influences the CD and is critical in achieving high (>100 mA/cm^2^) CD [98]. For example, hydrophobic gas diffusion layers (GDL) have been reported to increase the transfer of CO_2_ molecules toward the catalyst surface [95,96,97]. Ideally, effective porous catalyst layers should have both hydrophobicity to avoid flooding and hydrophilicity to help absorption of CO_2_ on catalysts [99,100,101]. Flooded pores completely eliminate gas channels within the catalyst layers and lead to high mass transport resistances for CO_2_ while dry pores are inactive due to the lack of aqueous electrolyte. Based on the capillary pressure, hierarchically porous structure with a mixed large pores and small pores could provide such conditions [102].

The challenges of stability can be reviewed at two levels and should be mitigated accordingly, i.e., system durability and component stability. At the system level, flow cells and MEA cells have several advantages over H-cells for CO_2_RR as they avoid CO_2_-solubility and mass transport limitations and thus can achieve higher CDs. However, their long-term durability has not been extensively studied yet. Several failure modes have been identified, such as formation and blockage of bubbles and carbonate precipitation on the electrode surface [17,19]. At the component level, there are several concerns, including catalyst degradation due to agglomeration and poisoning, electrode failures from anode oxidation and instability of layered cathodes based on GDL, and electrolyte changes of composition and pH [17]. A conventional gas diffusion electrode (GDE) typically consists of a gas diffusion layer (GDL), a microporous layer (MPL), and a catalyst layer (CL) [103]. The layered structure increases potential failures of delamination and instability of catalysts [17]. Free-standing three-dimensional hierarchically porous electrodes have the potential to overcome these issues, which is one of the motivations for writing this review paper.

This work reviewed recent progress in three-dimensional (3D) cathodes for CO_2_ reduction. In the literature, these cathodes have been either purposely constructed to be three-dimensional, or have had the potential to serve as three-dimensional [104,105,106,107,108]. 3D cathodes with hierarchically porous nanostructures offer many benefits, and are one of the most effective strategies to resolve the abovementioned challenges [109,110,111,112,113,114,115,116]. 3D cathodes may decrease the overpotential, increase the CD, and improve structural stability by

(1) Providing a larger number of electrochemical active sites;
(2) Facilitating transport of reactants and even changing product distribution or selectivity;
(3) Shortening ion and electron pathways;
(4) Avoiding delamination of layered structures by free-standing design.

**Figure 2:**
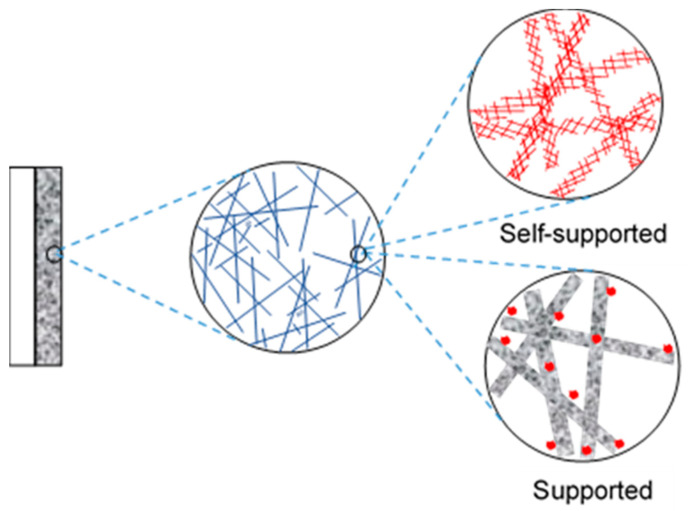
Hierarchical 3D catalyst layer with nanofibers as building blocks (red: catalysts).

Here we define 3D cathodes as electrodes with an interconnected macroporous architecture and nanostructured catalytic surfaces in three dimensions. Therefore, 3D cathodes not only represent an increase in thickness, but also an optimized hierarchical porous structure that improves mass transport and charge transfer while maintaining large active sites to address the challenges of low CD and high overpotential for electrochemical reduction of CO_2_. Ideal 3D cathodes can serve as freestanding electrodes without the need for additional GDL, which increase electrode performance and stability by avoiding additional interfaces and reduce time-consuming fabrication processes. The fabrication of 3D cathodes is reviewed in terms of different building blocks that are classified as self-supported catalysts (free-standing or non-supported), nanofiber-supported catalysts (1D blocks), graphene-supported catalysts (2D blocks), and porous materials-supported catalysts (3D blocks). Figure 2 illustrates a hierarchical 3D catalysts layer with 1D nanofiber as building blocks. In this work, all types of 1D block materials, such as nanotubes, nanowires, and nanoribbons, are considered part of the broader family of nanofiber materials. Two other families include graphene, represented by nanosheets and nanoplates; and porous materials, represented by reticulated vitreous carbon (RVC) foam and packed powders such as activated carbons.

Development of 3D cathodes is still in the early stages for electrochemical reduction of CO_2_, while 3D electrodes have been successfully used for other electrochemical systems such as fuel cells, batteries, and capacitors [116,117,118,119,120]. The perspectives of the present work comprise the first efforts to review the development of 3D cathodes and to provide suggestions for improving CO_2_ electrochemical reduction technology via nano- and microporous engineering.

## 2. Self-Supported Nanocatalysts for 3D Cathodes

In addition to the presence of numerous active sites in a catalyst/electrode structure, effective transport is required to reach higher current density and production rates. Inefficient transport results in thick electrochemical double layers and large local concentration gradients between the catalyst surface and reactants in the electrolyte and feed stream further increasing the resistance. This restricts the limiting current density and thus production rate.

3D electrodes may achieve both high active surface area for reactions and effective reactant/product transport. Higher production rates and efficiencies can be achieved when the active catalyst and electrode construction material are the same—self-supported 3D cathodes. This section reviews the electrocatalyst materials (building blocks) suitable for self-supported 3D cathodes as well as their fabrication methods and performance.

### 2.1. Nanocatalysts for CO_2_ Reduction

Among various types of electrocatalysts developed and studied for CO_2_ conversion, transition metals, heteroatom doped carbon and atomically dispersed metal/heteroatom doped carbon are amenable for the construction of 3D self-supported electrodes. This section briefly describes these classes of electrocatalysts, as well as their fabrication methods and catalytic properties for electrochemical CO_2_ conversion.

In transition metals, valence electrons of the d-band are close to the Fermi level. This unique property of transition metals enables them to overcome the intrinsic activation barriers required for efficient electrocatalytic activity [121]. The intrinsic catalytic properties of metals are easily tuned by alloying. Metals can also be easily processed via thermal and chemical routes to obtain specific surface morphologies/properties.

Although noble metals including Pt, Pd, Ir, and Rh are active and efficient for different electrochemical reactions, they are not considered to be selective/active for CO_2_ reduction. This is due to their small overpotentials for the competing hydrogen evolution reaction (HER). Silver and Au, on the other hand, have greater overpotentials for HER and are efficient and selective noble metal electrocatalysts capable of reducing CO_2_ to CO at low overpotentials and high Faradaic Efficiency (*FE*) [79,122,123]. However, due to the cost and scarcity of these metals, researchers have been increasing their focus on earth-abundant metals like Cu [124].

Copper is an active electrocatalyst for direct reduction of CO_2_ to alcohols and hydrocarbons in aqueous solutions. Depending on the electrolyte pH and other operating conditions, CO_2_ is first reduced at a certain cathodic potential to CO, which is weakly adsorbed on the Cu surface [125]. At more cathodic (negative) overpotentials, the adsorbed CO is further reduced to alcohols and hydrocarbons [125]. Copper is also the only transition metal known to produce C_2+_ products from CO_2_. However, Cu is not selective for a specific type of product and CO_2_ is reduced to a mixture of chemicals that need to be separated [126]. In addition, Cu is prone to poisoning, restructuring, and loss of activity [121].

Carbon nanomaterials as electrocatalysts benefit from several advantages including low cost, diverse and tunable morphology, high surface area, high electronic conductivity, excellent corrosion resistance and mechanical stability [1]. The other advantage of carbon-based electrocatalysts for CO_2_ electroreduction is their weak activity for parasitic HER. Pure carbon is not, however, active for electroreduction of CO_2_ and needs to be doped with other elements, including N, B, S and P [1]. When such elements replace carbon in the hexagonal lattice of graphite, graphene or nanotubes, the doped carbon material is referred to as “heteroatom-doped carbon”. The dopant elements alter the spin density and atomic charge of neighboring carbon atoms, which, in turn, generate active sites for CO_2_ electroreduction, particularly to CO [1,127].

Different types of heteroatom doped carbon including graphene [127,128], carbon nanotube (CNT) [129,130], nano-diamond [131], carbon nanofibers [132] and activated carbon (biomass-derived) [133] materials have been used as electrocatalysts for CO_2_ conversion. Morphology plays a critical role in determining selectivity of the heteroatom doped carbon self-supported 3D catalysts due to its effect on CO_2_ wetting and adsorption [134]. In particular, mesoporosity significantly affects selectivity and current density [134].

**Figure 3:**
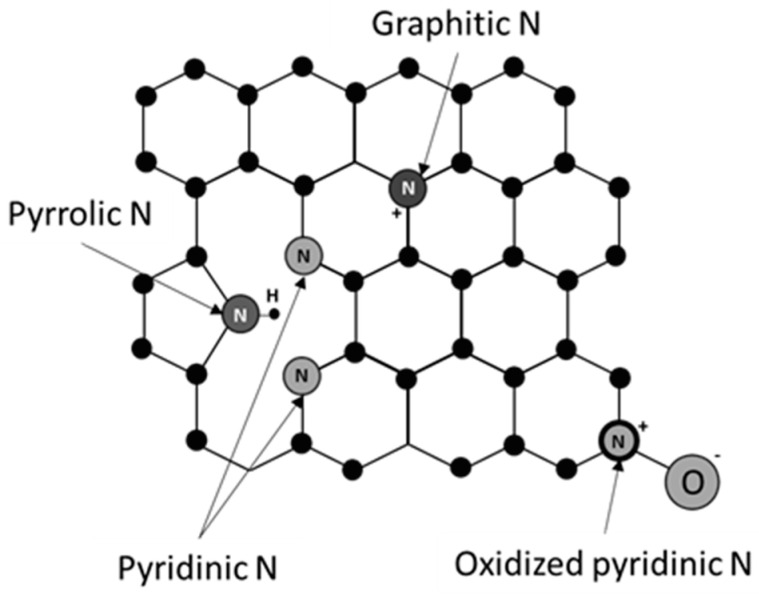
Positions of N atoms in carbon network of graphene [133,137]. Reproduced with permission from Elsevier, 2017.

Nitrogen and other heteroatoms can take multiple configurations in a carbon network. These include graphitic N, pyrrolic N, pyridinic N and oxidized pyridinic N (N-oxide) [133]. Figure 3 schematically shows these configurations. Density functional theory (DFT) calculations and experimental work show that pyridinic N is the most active configuration that offers the lowest free energy barrier for adsorbed COOH* formation among other N configurations [135]. This is due to the high energy of dangling N bonds leading to strong adsorption of the COOH* intermediate [136].

Dispersion of transition metal atoms in heteroatom-doped carbon materials can significantly enhance the catalytic activity. These catalysts are sometimes referred to as single atom catalysts (SAC). Nitrogen atoms in the carbon network stabilize metal atoms (M) in M-N_x_ (e.g., Ni-N_4_) coordination as active sites [138]. These catalyst materials have been shown to be selective for CO generation due to reduced energy barrier for adsorbed COOH* intermediate as in N-doped carbon catalysts [139]. Vacancies in lattice around the metal atoms play a critical role in catalytic activity of SACs [140].

### 2.2. Advantages of Using Self-Supported Catalysts

Electrochemical reactions need a triple-phase boundary (catalyst, electrolyte and reactants) in an electrode to take place. Conventionally, to provide the triple-phase boundary, powdered catalysts are mixed with an electronically conductive support, an ionomer (e.g., Nafion) and an optional binder (e.g., polyvinylidene fluoride (PVDF)) to form inks or pastes. The inks and pastes are subsequently applied to a current collector to form a practical electrode.

The catalyst support is a material that provides the required porosity for electrolyte access, transport of reactants to the catalyst surface (at triple-phase boundary) and removal of the products. A catalyst support is also responsible for electronically connecting the catalyst surfaces throughout the electrode volume to the current collector. The support material is not usually electrochemically active. The ionomer can act as a binder in addition to providing ionic conductivity within the electrode.

The self-supported catalysts with interconnected 3D pore structure can effectively transport reactants and products and provide electrical conductivity. The interconnected pore structure needs to be hierarchal to support both convection and diffusion mass transfer mechanisms. Therefore, the 3D self-supported catalyst should contain macroporosity (pore diameter > 50 nm), mesoporosity (2 nm < pore diameter < 50 nm) and microporosity (pore diameter < 2 nm). Such a hierarchical pore structure ensures a high surface area and takes advantage of the size effect of nanoparticles.

3D self-supported catalysts can be used in different levels including the catalyst layer (CL) or the gas diffusion electrode (GDE), depending on the reactor design. Unlike powder-based catalysts, self-supported catalysts do not need a binder and support and, therefore, offer the following advantages:

- Excellent electron transfer and electrical conductivity due to interconnectedness of their structure;
- Maximal use of the active surface area due to absence of binder and inactive support particles covering a portion of active sites; and
- Enhanced mechanical stability due to absence of weak binder-catalyst-support interfaces.

### 2.3. Fabrication Techniques of Self-Supported Catalysts

Metal foams with open cell, hierarchical structures have the potential for application as 3D self-supported electrodes. Commercial metal foams are produced through different routes, including melt blowing, powder metallurgy, template-directed methods and electrodeposition.

Gas bubbles in molten metal act as templates and create porosity upon solidification of the metal. Inert gases can be directly blown into the molten metal. Alternatively, gas bubbles can be generated within the molten metal by addition of foaming (gas releasing) agents.

In the powder metallurgy route, metal powder is mixed with a foaming agent (e.g., TiH_2_) and the mixture is compacted to produce a precursor (e.g., a disc) [141]. This precursor is then placed in a mold and heated in a furnace above the melting point of the metal. Once melted, the foaming agent releases the gas and the molten metal expands in the mold to produce the final product.

Open cell metal foams have been fabricated using reticulated polyurethane replica [142]. The replica is coated with a slurry of metal particles and a binder. Subsequently, the polymer replica is burned off. The product is then sintered at high temperatures to obtain a mechanically robust, free-standing foam.

Commercial metal foams have smooth surfaces and small specific surface areas, and are thus not suitable for electrocatalysis. The smooth foam surfaces need to be roughened by thermal and chemical treatments. Texturing and creation of nanostructures on the surface requires an appropriate surface treatment.

In addition to melt processing, electrodeposition is a versatile technique for producing hierarchally structured metal foams. Electrodeposition of metals at current densities (and overpotentials) higher than the limiting current density leads to a mass transfer-controlled condition. In aqueous electrolytes, this leads to domination of HER rather than metal deposition. Hydrogen bubbles start to nucleate and growth on the cathode surface as the result of HER. The bubbles, when stuck to the cathode surface, act as templates, and the metal crystals (dendrites) grow around them, producing a porous network of the deposited metal.

The other consequence of mass transfer-controlled deposition is formation of dendrites. Within the cathode double layer, metal crystals nucleate and grow toward the double layer/electrolyte interface, where the metal ions are more abundant and the diffusion distance is shorter. This results in dendritic electrocrystallization. Both hydrogen bubble-induced porosity and dendritic growth are essential for construction of self-supported 3D electrodes with a wide range of porosity from microporosity to mesoporosity and macroporosity.

The electrodeposited metal foams must have interconnected pore structures (open cell porosity) as well as sufficient and stable mechanical stability over long-term operation for practical applications as an electrocatalyst. The electrolyte chemistry and operating parameters must be adjusted to prevent formation of powdery and loose deposits. The bath chemistry plays a critical role in determining the deposit morphology. In particular, the presence of additives in small concentrations can drastically alter the morphology and affect the mechanical strength of electrodeposited metals.

In the electronics industry, additives with different functions are used to control the deposition behavior of Cu. The additives regulate the presence of cuprous (Cu^+^) intermediates at the cathode surface, which greatly affects the overpotential and kinetics of Cu electrocrystallization [143]. Electrodeposition additives are classified as inhibitors (also referred to as levelers or suppressors) and accelerators. In sulfate-based acidic Cu electrodeposition baths, inhibitors (e.g., polyethylene glycol (PEG)) interact with Cl^−^ and Cu^+^ ions and form a passivating film on the cathode surface that significantly reduces the deposition rate [144].

Accelerators (e.g., short chain disulfide or thiol molecules with a sulfonate-end group(s)), on the other hand, adsorb on the surface and increase the deposition rate by displacing the passivating film [144]. The ability of the accelerator for displacing passivating film greatly increases with increasing overpotential. This results in higher deposition rates on advancing concave regions, which is necessary for the superfilling of vias in printed circuits [145]. This dynamic completion between the inhibitor and accelerator may be beneficial for improving mechanical robustness of electrodeposited Cu.

**Figure 4:**
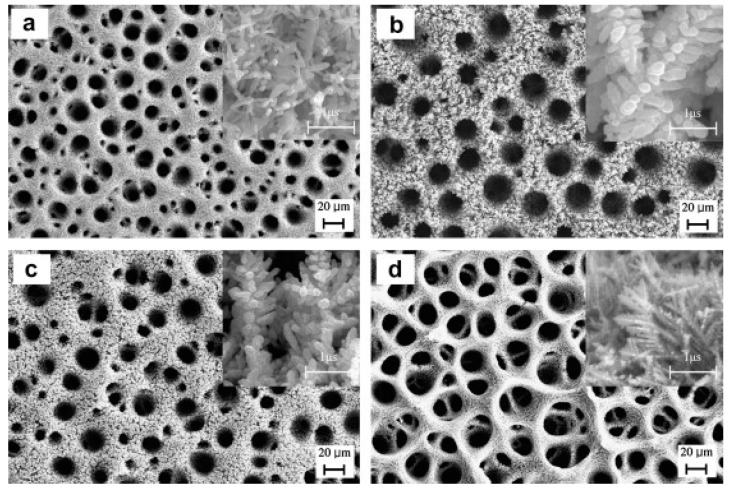
SEM images of electrodeposited Cu foams from additive-free (**a**), NH^4+^ (**b**), NH^4+^ and PEG (**c**), and NH^4+^, PEG and MPSA additized (**d**) Cu sulfate baths [146]; Reproduced with permission from Elsevier, 2008.

Kim et al. [146] studied the effect of NH^4+^, Cl^−^, PEG (inhibitor) and 3-mercapto-1-propane sulfonic acid (MPSA) on the morphology of electrodeposited Cu foams. The results showed that NH^4+^ increases the deposition overpotential for H_2_ and Cu and leads to a closed cell pore structure. Only in the presence of both inhibitor and accelerator was a 3D interconnected pore structure achieved. The pore walls were thinner, and the dendrites were much finer, but more compact than those obtained from additive-free bath and baths containing NH^4+^ alone or in combination with PEG. Figure 4 shows the scanning electron microscope (SEM) images of Cu foams deposited in the presence of different additives at 3 A cm^−2^ current density.

In addition to bath chemistry, operating parameters including overpotential, temperature, and electrolyte agitation affect the deposits’ microstructure. Higher overpotentials, lower temperatures and less agitation favor dendritic electrocrystallization. Higher overpotentials favor 3D growth and promote nucleation rate, leading to finer structures. Both bath chemistry and operating parameters need to be adjusted to achieve mechanically robust electrodeposited foams.

Optical lithography is a method for synthesizing templates of interconnected pore networks with controlled pore size for electrodeposition. In particular, proximity-field nanopatterning (PnP) is a method that can produce 3D templates with features down to 50 nm [147,148]. In this process, a substrate is coated with a photopolymer precursor by spin casting. A phase mask defining the depth and layout of the features is placed on the photopolymer-coated substrate, and the assembly is exposed to culminated light with a specific wavelength. The phase mask distributes the beam intensity in a 3D space within the photopolymer thickness (<15 µm) [148]. The regions exposed to the beam acidify and polymerize after exposure to heat (e.g., 75 °C for 5–10 min) [148]. A subsequent developing process dissolves and removes the unaffected photoresist in a solvent (e.g., propylene glycol monomethyl ether acetate). To prevent collapse due to capillary forces and retain the nano-sized features, the solvent is removed by CO_2_ supercritical drying [148]. The product is a nanopatterned polymer template suitable for electrodeposition.

Carbon-based self-supported 3D hierarchically structured electrocatalysts can be synthesized through pyrolyzing a solid polymer with 3D hierarchical pore structures (a membrane). Polymer foams and cellular structures can be synthesized using templating methods [134]. Polymer membranes have also been fabricated using phase inversion processes [149].

Templating is a practical route for fabrication of membranes with open cell 3D structures. Spherical particles of silica or polystyrene with controlled size and shape are commonly used as templates. Templating enables synthesis of polymer membranes with ordered structures and interconnected pores [150]. The templating particles are first assembled from a suspension into close packed crystal-like structures on a substrate. Then the gaps between individual particles are filled by a liquid polymer precursor, which is then polymerized. After polymerization, the template spheres can be chemically etched to produce a 3D porous membrane with interconnected, well-defined pore structures.

In the phase inversion process for synthesis of porous membranes, a polymer solution is cast on a substrate and immersed in a non-solvent bath (coagulation bath) where liquid–liquid phase separation occurs. In the coagulation bath, the diffusional exchange between solvent and non-solvent causes local concentration variations. This results in local supersaturation of polymer in solvent and its precipitation as a porous membrane [149]. The pore size and structure can be controlled by the rate of polymer precipitation and choice of solvent. Low and high precipitation rates result in symmetric and asymmetric pore structures, respectively [149].

For synthesis of 3D graphene foams, Ni foams are coated with graphene by a chemical vapor deposition (CVD) method [136,151]. A methane precursor decomposes at 1000 °C to H_2_ gas and graphene sheets which precipitate on Ni foam surfaces. To preserve the graphene 3D structure after removing Ni foam by chemical etching, a layer of poly(methyl methacrylate) (PMMA) is deposited on graphene-coated Ni foams [151]. The PMMA coating is removed by dissolution in acetone subsequent to Ni etching. The resulting graphene foam consists of interlocked graphene sheets with excellent electrical conductivity. Infiltration of graphene foam with poly(dimethyl siloxane) (PDMS) results in a stretchable porous sheet with excellent electrical conductivity [151].

Nitrogen doping of carbon nanomaterials can be achieved in situ by incorporation of N in polymer precursors [134,152,153]. Treating carbon materials at high temperatures with N or B sources is the main ex situ method [153]. Doping of carbon nanomaterials with N can be also be achieved by low-pressure NH_3_ plasma treatment [152,154]. Nitrogen ion implantation and annealing carbon nanomaterials in NH_3_ are the alternative routes for N doping [155,156]. Incorporation of metal atoms in a heteroatom doped carbon nanomaterial can be achieved through mixing polymer precursors with metal salts followed by polymerization and pyrolysis [157].

### 2.4. Cases of Applications for CO_2_ Reduction

Thermal oxidation of smooth Cu foam surfaces followed by reduction results in the formation of Cu nanowires and a significant increase in the active surface area. Raciti et al. [116] described a two-step thermal oxidation/reduction method to treat commercial Cu mesh for electrochemical CO_2_ reduction. Thermal oxidation of Cu mesh at 600 °C in air for 8 h resulted in growth of dense CuO nanowires. Metallic Cu nanowires were obtained through a subsequent reduction by either annealing in H_2_ or cathodic treatment. The Cu nanowires obtained through cathodic reduction showed substantial improvement in terms of high *FE* at low overpotentials for CO generation (i.e., 60% *FE* at 0.4 V and 1 mA cm^−2^) in comparison with oxide-derived and polycrystalline Cu.

In a later study, Raciti et al. [108] carried out thermal oxidation/reduction for a commercial Cu foam with an open cell structure (50 µm dia.). Three temperatures, 500 °C, 600 °C and 700 °C, were chosen for oxidation of Cu foam in air. Oxidation at 500 °C and 600 °C resulted in growth of dense nanowires and a mixed nanowire/porosity structure. Oxidation at 700 °C created only a porous structure.

Nanowire formation was believed to be the result of stress-driven, outward grain boundary cation diffusion. Oxidation of Cu results in a double layer oxide scale consisting of an inner Cu_2_O and outer CuO layer. Compressive stresses are generated within the CuO layer as the result of solid state transformation at the Cu_2_O/CuO interface that is associated with volume changes [158]. The stress gradient from the inner layer to the surface drives the outward Cu cation diffusion along the CuO grain boundaries and lead to acicular growth of CuO [158].

However, annealing at higher temperatures, e.g., 700 °C, may result in stress relief due to fast diffusion and facelifted lattice rearrangement, accommodating the stress [158]. In other words, the growth proceeds toward equilibrium where the system is allowed to minimize its surface to volume energies. The void and porosity formation at higher annealing temperatures can be attributed to outward diffusion of Cu cations and the Kirkendall effect.

The oxide structures on the Cu foam were reduced by potentiostatic cathodic treatment. Reduction of the oxide did not significantly alter the nanostructure’s morphology. Oxidation at 600 °C resulted in the largest electrochemical surface area (ECSA), as measured by cycling voltammetry in the non-Faradaic region. These specimens also showed the highest CO_2_ conversion rate (100 nM s^−1^ cm^−2^) in comparison with those oxidized at 500 °C and 700 °C. This was attributed to the presence of both nanowires at porosity for specimens oxidized at 600 °C. In comparison with a similar treatment for Cu mesh, the foams showed a 3-fold improvement in conversion rate.

The authors attributed the improvement in conversion rate to better mass transfer for foam electrodes. However, the comparison was made based on the geometric surface area. An accurate comparison requires calculating conversion rates based on ECSA. In other words, the maximum current used for CO_2_ reduction is higher for an electrode with higher ECSA in comparison with that with lower ECSA, but the same geometrical surface area. Once the data have been normalized for ECSA instead of geometric surface area, the advantage of foam (3D) over mesh (2D) can be determined. In addition, the polarization curves for all electrodes must be corrected for Ohmic resistance (IR correction).

**Figure 5:**
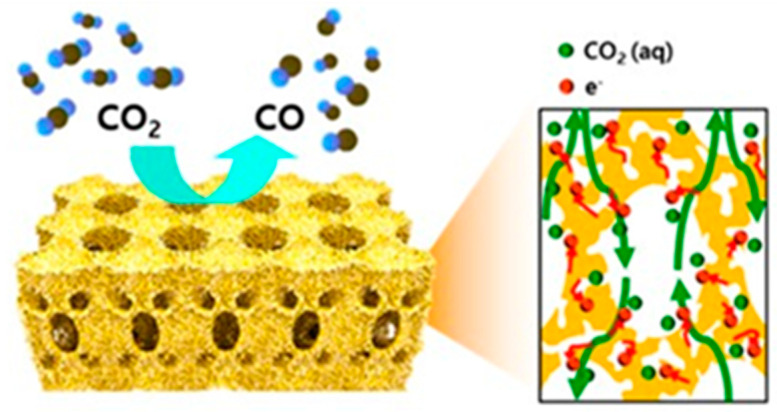
3D self-assembled Au electrode with 200–300 nm channels and 10 nm mesoporosity, synthesized by PnP and electrodeposition [104]. Reproduced with permission from PNAS, 2017.

Hyun et al. [104] studied the effect of ion and electron transport on electrochemical conversion of CO_2_ for 3D self-supported Au electrodes. Pure Au and Au–Ag alloys were electrodeposited on 3D templates fabricated through a PnP process. The template was removed by remote plasma etching after electrodeposition, leaving a free-standing 3D metal structure with 200–300 nm channels. For Au–Ag samples, dealloying in nitric acid results in dissolution of Ag and mesoporosity (~10 nm). Figure 5 schematically shows the structure of 3D self-assembled Au electrodes.

The dealloyed specimens exhibited 89% CO selectivity at −0.57 V and 6.8 mA cm^−2^ CO partial current density. The 3D structure of dealloyed samples increased the mass activity by four times in comparison with a flat electrode with the same microstructure.

For alloys with low melting point, gas releasing aqueous chemical reactions can be used for melting and foaming. An example of a low melting point alloy is the Field’s metal, which is a eutectic alloy with the composition of 32.5 wt.% Bi, 51 wt.% In and 16.5 wt.% Sn and a melting point of 62 °C. Allioux et al. [159] developed a method for the fabrication of catalytic metal foams using Field’s metal. The Field’s metal was mixed with NaHCO_3_ and glycerol, and the mixture was heated to above the melting point of Field’s metal under constant sonication. This process produces a suspension of liquid metal nanoparticles in NaHCO_3_ and glycerol solution. The addition of this suspension to aqueous hydrochloric acid solution resulted in endothermic reaction between NaHCO_3_ and HCl and CO_2_ gas evolution.

The CO_2_ gas bubbles acted as templates and simultaneously cooled the suspension. This resulted in sintering of metal nanoparticles in an open cell structure. The HCl solution etched the metal surfaces and texturized the surface. The characterization using X-ray photoelectron spectroscopy (XPS) showed that the foams fabricated in 0.1 M and 1 M HCl solutions at room temperature had a metallic core covered by a layer of Bi_2_O_3_ and nanocrystals of In_2_O_3_, SnO and SnO_2_. These foams were tested for electrochemical activity for CO_2_ reduction in 0.1 KHCO_3_ saturated with CO_2_. The foams were selective toward formate generation. The maximum *FE* was 80% at −0.8 V.

Klingan et al. [160] studied the activity and selectivity of Cu foams obtained by electrodeposition. The total pore volume of the electrodeposited foams were about 98%, regardless of the morphology. At low overpotentials, the ECSA was the only parameter affecting the specificity and partial current densities of the products. The foams with larger ECSA showed selectivity for CO and C_2_H_4_ at low overpotentials. The internal pore volume and morphology were not, however, related to these parameters. Klingan et al. [160] attributed this effect to the diffusion-limited proton supply to catalytic sites on Cu foam and alkalization of the surface, which limited the generation of H_2_ and CH_4_. Although partial current densities for both CO and C_2_H_4_ were a function of ECSA, a greater effect was observed for C_2_H_4_ as a result of increased ECSA. These investigators hypothesized that the simultaneous presence of CO dissolved in pores and the adsorbed *CO were the precursors for C_2_H_4_ generation.

Hursán et al. [134] studied the effect of porosity on activity and selectivity of N-doped carbon electrodes with well-defined pores. The self-supported electrode was made by a template method. Monodispersed, self-assembled silica particles in a monomer matrix were used as the membrane precursors. After chemical polymerization and carbonization, silica particles were chemically etched to create interconnected porosity. Three silica particle sizes, 13, 27 and 90 nm diameter, were selected for synthesis of the electrode. The pore volumes were the same for all electrodes. For comparison, an electrode without porosity was fabricated through the same process.

These electrodes were used for a systematic study of the effect of morphology on catalytic properties. There was a marked difference between porous and solid electrodes in terms of selectivity for CO generation over HER. Porous electrodes showed up to a 3-fold increase in selectivity. The electrode made with 27 nm silica template was the most selective. Hursán et al. [134] stated that the improvement mechanism was complex and attributed it to the changes in CO_2_ adsorption and wetting properties of the electrodes and other geometrical factors influenced by the pore size.

Wu et al. [136] theoretically (DFT calculations) and experimentally studied the electrochemical activity of N-doped 3D graphene foam. The foams were synthesized by CVD and doped ex situ after graphene deposition by thermal treatment in presence of solid, graphitic C_3_N_4_ at different temperatures. The maximum *FE* was approximately 85% for CO generation at −0.58 V overpotential, comparable with Au and Ag.

Zhao et al. [161] developed a method for fabrication of a 3D self-supported carbon paper (composed of CNT) with atomically dispersed Ni based on solid-state diffusion. A carbon/nitrogen precursor, i.e., melamine, was sprayed onto a Ni foil. Pyrolyzing the coated Ni foil at 1000 °C resulted in formation of C-N structure on the Ni foil. Nickel atoms from the foil surface diffused into the carbon vacancies of the coating. The diffused Ni in the form of nanoparticles catalyzed formation of NCNT on top of the C-N layer. The perpendicular arrangement of NCNT on the C-N layer produced a free-standing, flexible paper that peeled off from the Ni foil.

A subsequent acid leaching resulted in dissolution of exposed Ni nanoparticles. Leaching could not affect the Ni encapsulated in graphene layers. Homogeneous dispersion of Ni and N were observed on the grown CNTs. The electrode showed FEs larger than 90% in the potential range between −0.7 and −1.2). Zhao et al. [161] performed DFT calculations for a cluster of Ni (Ni309) and NiN_x_ sites to understand the conversion mechanisms. Reduction of CO_2_ to COOH* was found to be uphill for both Ni_309_ and NiN_x_ sites, in terms of free energies. Reduction of the intermediate COOH* to CO* was downhill for all sites. The main difference between Ni_309_ and NiN_x_ sites was determined to be desorption of CO*, which required larger free energy for Ni_309_. In contrast to Ni_309_, chemisorption of H* at the NiN_x_ site was thermodynamically unfavorable, indicating the importance of the singularity of the Ni atoms.

There is a large number of high-quality articles in the literature on catalyst development for CO_2_ electrochemical conversion. Some have shown high FEs at low overpotentials, particularly those with single atom dispersion with self-supported 3D structures. Many studies have concentrated on the deconvolution of mechanistic aspects of the developed catalysts in kinetic region and short term performance evaluation. Particularly for self-supported 3D catalysts or electrodes with complex pore size/structure, transport phenomena are critical [104].

Despite a few recent studies (e.g., [162,163]), it is rare to encounter articles focusing on durability for these catalyst materials. Most reported stabilities do not exceed a few hours of continuous operation. Obviously, electrochemical polarization results in changes in surface properties for many materials. It is important to study the aging phenomena for catalyst materials to understand the degradation mechanisms.

In addition, most studies used simple electrochemical cells with limited current densities far from the industrially accepted lower limit of 200 mA cm^−2^ [58]. In other words, catalyst materials are being evaluated for performance for the conditions that are different from those required for commercial electrolyzers [58]. Current density and transport phenomena have a significant effect on reaction rate, selectivity, reactivity and durability of electrodes. Therefore, it may be the time to focus on integration of different catalysts into electrodes and developing a reactor that is more representative of conditions to be experienced in commercial electrolyzers.

This is essential for relevant characterization of catalyst materials and optimization of the performance. In particular, polymer membrane electrolytes (either proton-exchange or anion exchange membranes) allow markedly higher current densities (i.e., production rate) than liquid electrolytes. The knowledge gained over the course of PEM fuel cell (and water electrolyzer) development may be used to develop high-performance reactors for CO_2_ conversions.

## 3. Nanofiber-Supported Nanocatalysts for 3D Cathodes

Supported catalysts are capable of assisting and improving the catalyst performance in various ways, such as increasing the catalytic activity via better dispersion and interaction with support, increasing the stability by preventing particle aggregation and poisoning, and decreasing the cost by using catalyst lower loading compared to unsupported bulk catalysts [164]. Hence, the choice of support material is vital and highly influential in determining the behavior, performance, longevity and cost effectiveness of the catalyst and the overall CO_2_ reduction system. Similar to the prerequisite properties for cathodes (except required catalytic activity), ideal support materials also interact with catalysts to enhance catalytic efficiency and selectivity [165]. Compared with conventional support materials, nanoparticle matrices offer a higher catalyst loading capacity due to their very large surface areas [166,167]. With novel nanomaterials technologies, the methodologies for preparing nanomaterials-supported catalysts are also different compared with the conventional synthesis of supported catalysts, such as impregnation and co-precipitation methods [168,169,170]. Various nanomaterials have been widely investigated as catalyst supports for electrochemical devices. These include carbon-based materials such as activated carbons, carbon nanotubes (CNTs), carbon nanofibers (CNFs), carbon cloths (CC), and graphene and non-carbon-based materials such as oxides and polymers [164]. One-dimensional nanomaterials offer larger surface areas, extra surface-active sites, and better permeability that are required as support materials for CO_2_RR. One-dimensional nanomaterials are synthesized by various methods, including template-based synthesis, vapor-phase growth, solution-based growth or electrospinning, and other innovative techniques [170,171,172]. These preparation routes provide controllable synthesis of one-dimensional nanomaterials with different morphologies, porosities and inner structures. As evidenced in the discussion below, nanofibers and other one-dimensional nanomaterials such as nanowires, nanotubes, nanorods, nanobelts, and nanoribbons have been used as support materials for electrocatalysts in the construction of 3D cathodes for CO_2_ reduction. This section reviewed selected studies of 3D cathodes based on polymer, CNTs, CNFs, and oxide nanofibers for CO_2_ reduction. While some of the progress is promising, most of the work to date has not yet demonstrated the advantages of 3D cathodes, leaving room for inspiration and future improvement.

### 3.1. Polymer Nanofibers

Polymers, particularly conducting polymers that conduct protons and electrons, have the potential to be employed as an ideal catalyst support for electrochemical applications [164]. Recently, a 3D freestanding cathode (i.e., no need for GDL) to enhance CO_2_ mass transport was demonstrated based on polyvinylidene fluoride (PVDF) nanofibers with Sn/Cu as catalysts (Sn/Cu-PVDF) [14]. PVDF, displaying good chemical and thermal stability, are widely used in filtration and separation equipment and as separators and ionic conductors in lithium ion batteries.

**Figure 6:**
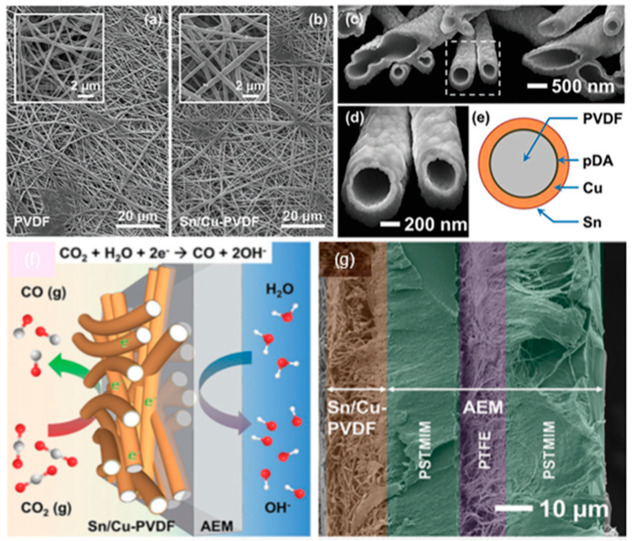
Top-view SEM images of (**a**) a PVDF membrane and (**b**) a Sn/Cu-PVDF electrode. (**c**) Cross-sectional SEM image of Sn/Cu-PVDF nanofibers and (**d**) high-resolution SEM image from the selected region in (**c**). (**e**) Scheme of the composition of each layer of a Sn/Cu-PVDF nanofiber. (**f**) Scheme and (**g**) cross-sectional SEM image of a Sn/Cu-PVDF/AEM assembly used for electrocatalytic reduction of gaseous CO_2_ [14]. Reproduced with permission from Wiley, 2019.

The PVDF nanofibers are fabricated by electrospinning and their surface is activated by grafting a self-assembled polydopamine (pDA) layer to provide nuclei for electroless Cu deposition. Then Sn is decorated on the Cu surface by the electrochemical underpotential deposition (UPD). A Sn/Cu-PVDF nanofiber electrode, acting as a freestanding gas diffusion electrode (GDE), is pressed onto an anion exchange membrane (AEM) at 125 °C. Figure 6 shows a cross-sectional SEM image of a Sn/Cu-PVDF/AEM assembly. The AEM used in this work has a three-layer structure with a carrier of expanded polytetrafluoroethylene (PTFE) sandwiched between two polystyrene tetramethyl methylimidazolium (PSTMIM) membranes.

It has been demonstrated that Sn/Cu-PVDF freestanding GDEs have *FE* values of above 80% for CO and CD of up to 104 mA cm^−2^ at −1.2 V vs. RHE. The loading of Sn on the surface of Cu was found to be critical for achieving high selectivity for CO_2_RR to CO from this and previous work [173,174]. When the loading of Sn on the Cu surface exceeds the optimal value, the Sn/Cu catalysts behave similarly to a Sn electrode, resulting in a different reduction product of formate [175,176].

The 3D freestanding electrode maintains an average *FE* for CO over 84.9% at −1.0 V vs. RHE for a period of 135 h. The loss of CD is mainly attributed to the increase of the ohmic resistance in the MEA. The influence of CO_2_ mass transport on the CD was compared between two electrodes with similar loading of catalysts but different permeability. One was a 2.5-μm-thick Sn/Cu-polycaprolactam (Cu/Sn-nylon) electrode with an intrinsic permeability of 3.5 × 10^−15^ m^2^ and the other was an 8-μm-thick Sn/Cu-PVDF electrode with intrinsic permeability of 1.8 × 10^−14^ m^2^. With a less efficient CO_2_ mass transport, the Sn/Cu-nylon electrode reaches lower CO partial CD, of 63.7 mA cm^−2^, compared to the CD of 104 mA cm^−2^ at −1.2 V from more porous Sn/Cu-PVDF electrode.

### 3.2. Carbon Nanotubes and Nanofibers

While polymer nanofibers as building blocks have demonstrated potential for CO_2_ reduction, there are still some concerns regarding practical applications, such as stability in aqueous conditions and electrical conductivity. Carbon-based materials offer many advantages as supports of catalysts, including satisfactory cost, structure diversity, good electrical and thermal conductivity, mechanical strength and lightness, and easy end-of-life recovery of precious metals resulting in a low environmental impact. CNFs and CNTs are promising support materials, as they have the combination of large surface areas with unique properties of carbon [177]. Various synthetic methods for producing CNFs and CNTs have been reported, while catalytic chemical vapor deposition (CCVD) and electrospinning methods have been the dominant and effective techniques for large-scale production [178,179]. Nanocatalysts can be co-synthesized or deposited on CNFs and CNTs using a variety of deposition methods including impregnation, ultrasound, sputter deposition, precipitation, and electrochemical deposition [164]. However, carbons are chemically inert in nature, and surface treatment is necessary for deposition of metallic catalyst particles.

**Figure 7:**
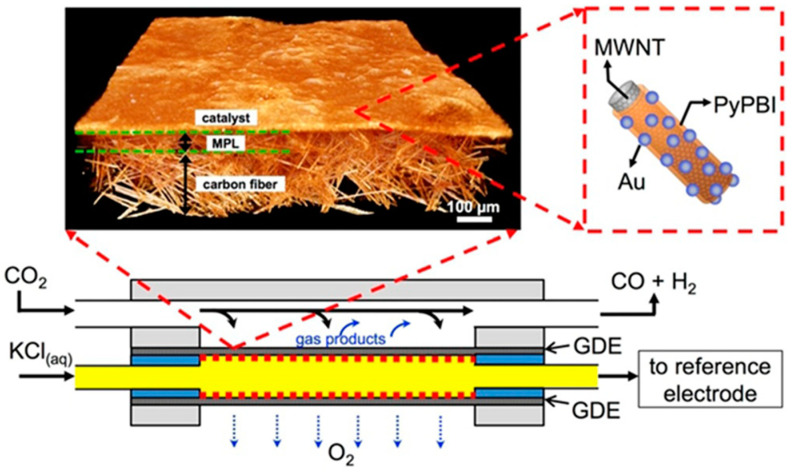
Schematic representation of the Au catalyst supported on polymer-wrapped multiwall carbon nanotubes (MWNT/PyPBI/Au) microfluidic electrolysis cell [181]. Reproduced with permission from Wiley, 2017.

CNTs have been widely studied as substrates of catalysts for CO_2_RR in recent years [180]. Recently, a catalyst of Au nanoparticles supported on polymer-wrapped multi-walled carbon nanotubes (MWNTs) as the cathode was prepared and evaluated in a microfluidic electrolysis cell as shown in Figure 7 [181]. Pyridine-containing polybenzimidazole (PyPBI) was used to provide nucleation sites for the in situ growth of metal nanoparticles. These polymers are not electrically conductive or catalytically active. Therefore, the polymer coatings must be thinner than 1 nm thick to ensure sufficient electronic contact between the MWNTs and metal nanoparticles via quantum tunneling [182]. The MWNT/PyPBI/Au cathodes were synthesized in a two-step process. The MWNT/PyPBI catalyst support were prepared by suspending the MWNTs in a solution of PyPBI in N,N-dimethylacetamide (DMAc), followed by filtering, rinsing, and drying under vacuum. Then, Au nanoparticles were grown in situ on the surfaces of the catalyst supports.

The cathodes with different configurations were studied and compared, including unsupported Au, carbon-black (CB)-supported Au (CB/Au), and Au on support of PyPBI-wrapped CB (CB/PyPBI/Au). The MWNT/PyPBI/Au cathode shows a partial CD of 160 mA cm^−2^ for CO production at a potential of −1.78 V vs. Ag/AgCl and *FE* of 60%. In comparison, the CB/PyPBI/Au and CB/Au cathodes reach a partial CD for CO of 90–100 mA cm^−2^ at similar electrode potentials, while all other cathodes exhibited significantly lower CD. The catalytic performance enhancements observed are largely attributed to the increase in specific electrochemically active surface area (ECSA). The MWNT/PyPBI/Au cathodes have the highest ECSA of 23 m^2^ g^−1^ Au, then the others decreased in the order CB/PyPBI/Au, CB/Au, and unsupported Au, which corresponded qualitatively with the trends in the observed relative partial CD for CO.

**Figure 8:**
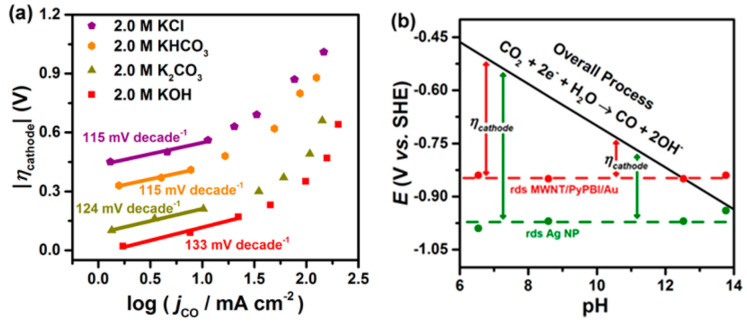
(**a**) Tafel slopes for the electroreduction of CO_2_ to CO using MWNT/PyPBI/Au as cathode and IrO_2_ as anode with different electrolytes. (**b**) E−pH (Pourbaix) diagram for the electroreduction of CO_2_ to CO (rds MWNT/PyPBI/Au and rds Ag NP denote the rate-determining step for these catalysts, respectively) [183]. Reproduced with permission from ACS Publications, 2018.

The MWNT-supported Au cathodes were further studied for CO production by replacing 1.0 M KCl electrolyte with 2.0 M KOH electrolyte, achieving a CD of 158 mA cm^−2^, overpotential of 0.94 V vs. RHE and *FE* of 49.4% [122]. The performance was stable for at least 8 h. Electrolyte ions and concentration was found to play a crucial role to improve overpotential and CD. The effect of KOH electrolyte in comparison to K_2_CO_3_, KHCO_3_, or KCl electrolytes with different concentrations are shown in Figure 8a. Anions were found to play an important role with respect to reducing the onset potential of CO. The values of onset potentials increased with different anions in the order of OH^−^ < HCO_3_^2−^ < HCO_3_^−^ < Cl^−^. In a previous study, it was demonstrated that CD of CO were improved by several fold when electrolyte concentrations were changed from 0.5 M to 3.0 M [183,184]. With Ag nanoparticles dispersed in Nafion on GDL (Sigracet 35 BC electrode, Ion Power Inc., New Castle, DE, USA) and 3.0 M KOH as electrolyte, CD of CO was as high as 440 mA cm^−2^ with an *FE* of 42% and overpotential of 1.1 V vs. RHE. However, at such high concentrations of KOH, CO_2_ would be consumed in the electrolyte and converted into carbonate/bicarbonate. Electrochemical impedance spectroscopy showed that both the charge transfer resistance and the cell resistance decreased when moving from a 0.5 M to a 3.0 M KOH electrolyte [183]. The onset cathode potentials, kinetic isotope effect, and Tafel slopes indicate the low overpotential production of CO in alkaline media to be the result of a proton or pH-independent rate-determining step (i.e., electron transfer controlled) in contrast to a pH-dependent overall process as shown in Figure 8b. Additionally, the equilibrium potential associated with the formation of CO_2_^−^ (ads) (−1.9 V vs. SHE) is much more negative than the onset potential of −0.84 to −0.85 V vs. SHE and −0.94 to −0.99 V vs. SHE for CO production on MWNT/PyPBI/Au and Ag nanoparticles, respectively. The large potential differences are attributed the electronic structure of the metallic catalysts, which determine their catalytic properties. The onset potential for CO production on MWNT/PyPBI/Au is more positive (better performance) in comparison to the value for Ag nanoparticles.

In addition to a more complex structure with greater surface area, MWNTs exhibit advantages over single-walled (SWNTs) and double-walled carbon nanotubes (DWNTs), such as ease of mass production, low product cost per unit, and enhanced thermal and chemical stability [185,186]. MWNTs as building-blocks for cathodes have great potential for effective CO_2_RR, and MWNTs with different catalysts have been reported. When cathodes with catalysts of tin oxide particles supported on MWNTs (SnO_x_/MWNT) with stainless steel mesh were prepared by both an in situ and a hydrothermal method, liquid phase electrolysis carried out in 0.1 M KHCO_3_ yielded an *FE* of 64% for formate formation and CD of ~5 mA cm^−2^ at 1.4 V vs. SCE for 20 h [187]. There is little difference in the electrocatalytic selectivity and activity from different morphologies of the particle agglomerates. However, with the cathode of SnO_2_/MWNT prepared by wet impregnation with carbon paper as support, liquid phase electrolysis carried out in 0.5 M NaHCO_3_ yielded *FE* of 27.2% and CD of 80 mA cm^−2^ at a potential of −1.7 V vs. SCE [188].

Carbon nanotube aerogel, a new kind of three-dimensional (3D) porous material with high specific surface area, good mechanical stability and superior conductivity, has great potential in many applications [189,190]. A 3D hierarchical porous structured carbon nanotube aerogel supported Sn spheroidal particles on carbon cloth (Sn/CNT-Agls/CC) cathode for electrochemical reduction of CO_2_ to formate has been reported [191]. The 3D Sn/CNT-Agls electrocatalyst was fabricated through a wet chemistry process followed by freeze-drying and calcination reduction under an H_2_ atmosphere. The as-prepared Sn/CNT-Agls has a specific surface area of 71.3 m^2^ g^−1^, average pore size of ca. 26.5 nm, and density of 16.2 mg cm^3^. A maximum *FE* of 82.7% for formate production has been achieved with a CD of 24 mA cm^−2^ at −0.96 V vs. RHE in 0.5 M KHCO_3_ for 5.6 h. As a comparison to the cathode of carbon nanotube-supported Sn spheroidal particles (Sn/CNT) on carbon cloth (Sn/CNT/CC) with a specific surface area of 71.3 m^2^ g^−1^, the CD of the Sn/CNT-Agls/CC electrode is three times higher than that of the Sn/CNT/CC electrode. The superior performance is most likely caused by the high specific surface area, multiple catalytically active sites and excellent conductive 3D hierarchical structure of the Sn/CNT-Agls/CC electrode.

Carbon materials have been widely used as support materials while pure carbon materials are inactive towards electrochemical reduction of CO_2_. However, it has been demonstrated that the carbon atoms around single atom centers can be activated and behave as catalysts [32]. Heteroatom doping, in particular, N-doping of carbon nanotubes (NCNT), has exhibited catalytic activity for CO_2_ reduction to CO [130,192]. Polyethyleneimine (PEI) overlayers can act as CO_2_ absorbents via van der Waals forces and have been shown to enhance the catalytic performance (PEI-NCNT) compared with NCNT and CNT [192]. In most cases, N-doped carbon electrocatalysts are developed in the form of powders and then bound with Nafion or PVDF as electrodes, suffering from a low productivity for the CO_2_ reduction [193]. A 3D hierarchically porous N-doped carbon membrane (HNCM) with CNT frame (HNCM/CNT) as binder-free electrode has been developed [193]. The cathode construction was performed via formation of a film membrane from CNT and polymer solution followed by pyrolysis of the porous polymer/CNTs at 900 °C in N_2_. The prepared porous carbon membranes were used directly as freestanding electrodes without the need for a GDL. In these membranes, the macropores provide mass transport highways while the mesopores and micropores provide a large surface area and high population of spatially accessible electroactive sites for CO_2_RR. The *FE* for the production of formate was 81% at −0.8 V vs. RHE at CD of 8 mA cm^−2^, with a electrochemical stability over 36 h at overpotential of −0.9 V in 0.1 M KHCO_3_. The 3D HNCM/CNT cathodes offer low overpotential and high *FE* compared to HNCM cathode (0.9 V, 32%) and other configurations of cathodes.

As doped carbons become catalytically active, nitrogen-doped CNFs (NCNFs) have been explored as potential support materials to promote interfacial interaction with catalysts for the CO_2_RR [165]. Nitrogen doping can modify the electronic structure of graphitic carbons and lead to increased electronic conductivity, high surface energy, and tunable chemisorption ability favoring electrochemical reactions [194,195]. Therefore, different types of catalysts with NCNF supports could lead to enhanced catalytic activity of different products of CO_2_RR. For example, a cathode with Co_x_Ni_1−x_ nanoalloys supported by NCNFs (Co_x_Ni_1−x_/NCNFs) were constructed and regulated via electrospinning procedures followed by pyrolysis [196]. The cathode of Co_0.75_Ni_0.25_/NCNFs showed *FE* of 85.0% for CO and a CD of 13.4 mA cm^−2^ at −0.9 V vs. RHE in 0.5 M NaHCO_3_. The *FE* of 85.0% is superior to most of the current noble metal free electrocatalysts, this selectivity was attributed to the modulated electronic configuration explained by DFT calculations. Additionally, via an electrospinning technique coupled with a pyrolysis process, cathodes were developed with Sn-modified NCNT (Sn-NCNT) hybrid catalyst [165]. The cathodes drive formate formation with a CD of 11 mA cm^−2^ and a *FE* of 62% at an overpotential of 0.69 V vs. RHE in 0.5 M KHCO_3_. However, atomically dispersed Sn with NCNTs promotes conversion of CO_2_ to CO, with an *FE* of 91% at an overpotential of 0.49 V after the Sn particles were removed from Sn-NCNT via acidic leaching. The interaction between Sn and N in NCNTs may play a role in tuning the catalytic activity and selectivity of these two products of CO_2_RR [129]. The local electronic environment from the supporting materials affects the electrocatalytic activity of Sn was observed by other researchers, as well [197,198]. Graphene-sheet-supported Sn nanoparticles exhibited enhanced electron donation and thus facilitated formate formation, compared with carbon-black-supported Sn. Understanding of the reaction kinetics involved in these reactions requires further mechanistic studies.

Different from CNFs and the CNTs matrix, commercially available carbon cloths (CCs) have a 3D network composed of carbon fibers with a diameter of 7–10 mm. CCs exhibit outstanding mechanical strength, light weight, and flexibility with high electrical conductivity. However, their low surface area (around 5–8 m^2^ g^−1^) and lack of active sites on their surface limit their direct applications as electrode materials [199]. A flexible 3D hierarchical structured cathode composed of mesoporous SnO_2_ nanosheets on carbon cloths (SnO_2_/CCs) have been studied for CO_2_ reduction to formate in 0.5 M NaHCO_3_ [200]. The SnO_2_/CCs cathodes were fabricated via a combination of hydrothermal reaction and calcination. Formate was produced with a *FE* of 87.2% and CD of ~45 mA cm^−2^ at an overpotential of 0.88 V vs. Ag/AgCl. The highly porous hierarchical structure provides a large surface area and facilitates charge and mass transfer. Various strategies have been developed to activate carbon fibers with increased porosity for further applications, including chemical, physical, electrochemical, and synthesis methods [201,202]. Either uniformly or hierarchically porous carbon fibers (PCFs) have been created. Activated carbon fibers (ACFs) that typically have diameters of ca. 10 mm, contain slit-shaped pores and have surface areas of 1400–1500 m^2^ g^−1^, have been promising support materials. This type of support, with a pronounced peak in the pore size distribution at ca. 2 nm works very effectively in the case of small catalytic particles [203,204,205]. Nanometer-scale pores generate effective pressures over 20 mPa, and thus enhance rates for certain reactions. An increase in the catalyst dimensions results in a decrease in the benefit; the so-called nanospace effect [206,207]. Therefore, ACFs as electrodes offer the advantages of large surface area and elevated pressure without the need for energy-wasting pressurization. The combination of organometallic catalysts with nitrogen containing macrocyclic ligands and ACFs as support materials may offer benefits to achieve high efficiency for CO_2_ electroreduction. Several porphyrin and phthalocyanine transition metal complexes with different sizes supported on activated carbon fibers with GDL as cathodes have been investigated and compared with catalysts on activated carbons (ACs) with surface area 2000 m^2^ g^−1^ [205]. The cathode was fabricated by mixing synthesized catalysts/ACFs with PTFE/acetylene black and applying to a GDL. The cathode with catalysts on ACFs yielded CO with an *FE* of 70% and CD of 70 mA cm^−2^ at −1.5 V vs. SCE in 0.5 M KHCO_3_ solutions. The catalyst with smaller complex sizes supported on ACFs showed better performance than those supported on ACs. Conversely, the larger complex catalysts supported on ACs showed better performance.

### 3.3. Oxide Nanofibers

**Figure 9:**
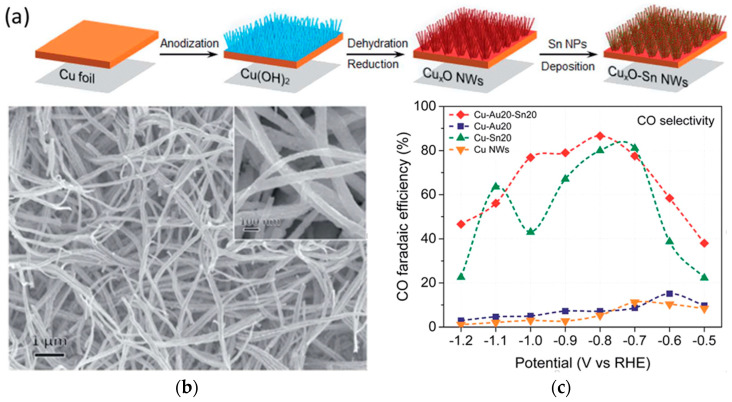
(**a**) Illustration of the fabrication process of Cu_x_O–Sn NWs; (**b**) typical SEM images of Cu_x_O–Sn20 NWs with different magnifications; (**c**) potential-dependent CO *FE* (Sn20 representing Sn electroless deposition for 20 s) [207]. Reproduced with permission from RSC, 2016.

Oxide nanofibers have also been proposed as a support material for CO_2_RR. However, there remains debate within the community as to the stability of oxides under reducing potentials. Oxides have the ability to facilitate CO_2_ adsorption and provide potential metal-oxide synergistic effects to enhance catalyst performance. A 3D cathode based on copper oxide nanowires (NWs) with decorated Sn nanoparticles on surface (Cu_x_O-Sn NWs) has been reported. The 3D cathode is fabricated on Cu foil by anodizing, dehydration reduction and electroless deposition of Sn, as shown in Figure 9a,b. The cathodes were able to reduce CO_2_ to CO with an *FE* of 90% and CD of 4.5 mA cm^−2^ at an overpotential of 0.69 V vs. RHE in 0.1 M KHCO_3_. As a comparison (Figure 9c), addition of Sn to Cu_x_O NWs electrode significantly enhanced performance of *FE* for CO over the other electrodes. It can be seen from the previous discussion, Cu catalysts on CNTs promote formation of CO while Sn catalyze production of formate [175,176,191]. However, the major product becomes CO for Sn on Cu_x_O in this study. Since selectivity is closely related to the reduction mechanism with different reaction pathways leading to different products, the synergistic interaction between the Sn and Cu_x_O seems to enhance *FE* and also to significantly alter the reaction pathways. It is well known that the reduction process is strongly affected by experimental conditions, such as the materials and structures of the catalysts and/or electrodes, electrode potential, electrolyte composition and concentration, buffer strength, pH, CO_2_ concentration, temperature, and pressure [208,209,210]. Many studies have demonstrated that oxides facilitate CO_2_ adsorption or the metal-oxide synergistic effect to enhance catalyst performance. For example, the Au–CeO_2_ interface enhances the conversion of CO_2_ to CO by increasing the binding strength of *COOH and easing CO_2_ activation [211].

Very recently, a 3D cathode based on network of CNFs embedded with Cu and CeO_x_ nanoparticles (Cu/CeO_x_-CNFs) was presented [212]. The cathodes were fabricated by nanofiber formation of electrospinning precursor solution of Cu, CeO_2_, and polyvinylpyrrolidone (PVP) on a nickel net and then carbonized at 800 °C under N_2_ atmosphere. In a flow cell configuration, a CD of 100 mA cm^−2^ and a *FE* of 59.2% for CO at −0.60 V vs. RHE in 1.0 M KOH were achieved. Addition of ceria was found to enhance the activity and CO-selectivity as well as decrease the selectivity of HER. The Cu–CeO_x_ interface in such an architecture offers synergistic geometric and electronic effects which optimize the adsorption strength of reaction intermediates for the electroreduction of CO2 to CO. Similarly, a cathode of Cu/SnO_x_ nanoparticles supported on CNTs (Cu/SnO_x_–CNTs) has also demonstrated enhanced catalytic performance influenced by metal/oxide interactions [213]. The catalysts of Cu/SnO_x_ were synthesized from precursor solution with CNTs and freeze-drying, and then applied on carbon fiber paper. SnO_x_ alters the catalytic behavior of Cu in the Cu-rich regime, while Cu modifies the catalytic properties of SnO_x_ in the Sn-rich regime. The Cu/SnO_x_−CNTs cathode containing 6.2% of SnO_x_ converts CO_2_ to CO with a *FE* of 89% and a CD of 11.3 mA cm^−2^ at −0.99 V vs. RHE in 0.1 M KHCO_3_ (pH 6.8), compared to the Cu−CNTs cathode with ethylene and methane as the main products of CO_2_ reduction. The Cu/SnO_x_−CNT cathode containing 30.2% of SnO_x_ reduces CO_2_ to formate with a *FE* of 77% and a CD of 4.0 mA cm^−2^ at −0.99 V, in contrast with the SnO_x_−CNT cathode, which converts CO_2_ mainly to formate with a *FE* of 48%.

Nanofibers as building blocks for 3D cathode construction have been widely studied as a cost-effective method and have shown promising results for increasing mass transport with macro-porous architecture and large surface area as sites for CO_2_RR. In particular, the feasibility of 3D freestanding cathodes has been demonstrated successfully without the need for additional GDLs. Nanofibers as building blocks for 3D cathode construction also have the advantage of fabricating catalysts with support materials and co-catalysts or catalyst-promoting materials such as selected oxides simultaneously by electrospinning processes. However, competitive performance has not yet been fully demonstrated due to the complex influencing factors. There remains a lack of studies for large-scale 3D cathodes, and even fewer studies that combine 3D cathodes with optimized conditions for optimal performance in a reactor system, as the majority of research has focused on the performance evaluation of catalysts. Novel nanofiber materials and related fabrication approaches for the construction of 3D cathodes are still needed with proven catalytic performance and long-term stability.

## 4. Graphene-Supported Nanocatalysts as Cathodes

Graphene is one the most effective components of electrocatalysts for different electrochemical processes, including the CO_2_ electrochemical reduction (CO_2_RR). One of more cost-effective and widely used cathode materials for CO_2_ electrochemical reduction are two-dimensional (2D) graphene-based materials. The solvent evaporation method has been used for the deposition of a catalytic film on the 2D graphene substrate, resulting in low wettability and a high number of active sites. However, the performance of 2D graphene-based catalysts for CO_2_RR can decrease due to the formation of irreversible agglomerates due to the strong the strong π–π bonds and van der Waals interaction between graphene sheets. This agglomeration may result in the reduction of the specific surface area and the adsorptivity of these electrodes towards CO_2_, porosity, and electrolyte penetration into catalysts [214,215,216,217]. The decrease in CO_2_ adsorption on self-agglomerated 2D graphene-based materials result in a reduced rate of CO_2_RR.

To mitigate these technical issues with 2D graphene, free-restacking three-dimensional (3D) graphene-based materials (hydrogels (HG), foam, sponges) have been proposed as electrodes or electrode supports for CO_2_RR prepared by through chemical reduction [217], the template method [218], and hydrothermal methods [219].

These electrodes have tunable porosity, high surface area, low cost, and 3D conductive pathway [220,221,222,223], e.g., HGs have porous structures containing H_2_O, providing greatly improved wettability and electrolyte penetration. Moreover, 3D porous conductive pathways in graphene facilitate gas diffusion. Active sites for CO_2_RR can be integrated into the 3D GH using various functional groups such as carboxyl (–COOH) and hydroxyl (–OH) groups and non-metal doping (N, S). The modification of 3D graphene with catalytically active coating of noble or non-noble metals greatly enhance the rate of CO_2_RR. All 3D graphene-based dimensional materials are divided into four groups:

- Metal-free 3D graphene network (3DGNW);
- Metal-free 3D graphene nanocompositions (3DGC);
- Non-noble metal 3D graphene-based nanocomposite (NNM 3DGC); and
- Noble metal 3D graphene-based nanocomposite (NM 3DGC).

### 4.1. Metal-Free 3D Graphene Network (3D-GNW)

**Figure 10:**
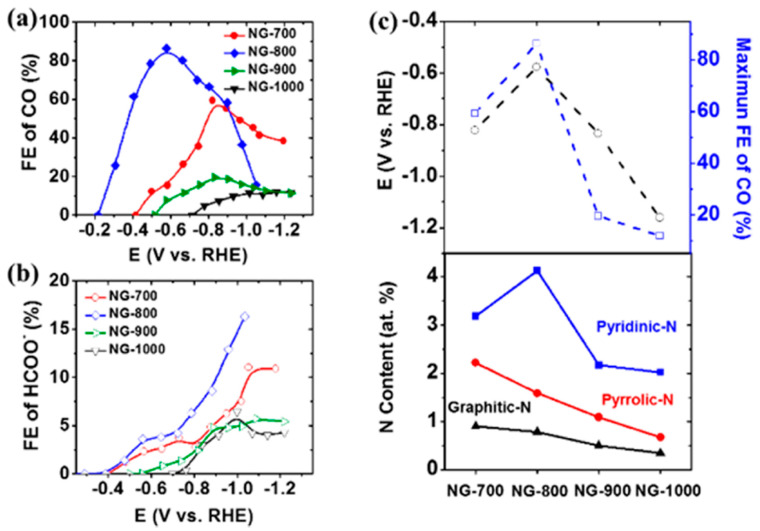
Comparison of the electrocatalytic activities of nitrogen-doped graphene with doping temperature ranging from 700 to 1000 °C. (**a**) Faradaic efficiency of CO versus potential. (**b**) Faradaic efficiency of HCOO- versus potential. (**c**) Maximum Faradaic efficiency of CO and its corresponding potential versus N functionality [136]. Reproduced with permission from ACS Publications, 2016.

This section briefly presents the metal-free 3D graphene network (3DGNW), as the topic has already been discussed in Section 2.3 and Section 2.4. 3D-GNWs include non-metal (N, S, B,)-doped 2D graphene [224]. The 2D graphene sheets in electrodes agglomerate due to the strong π–π bonds and van der Waals interactions, which significantly decrease their specific surface area. The main strategy to avoid this is the incorporation of nanoscopic 2D graphene sheets into the macroscopic 3D network. Noble metals, e.g., gold and silver, have demonstrated a high electrocatalytic activity for CO_2_RR into CO, but they have poor durability and cost efficiency. To remedy this, a catalyst based on nitrogen-doped 3D graphene foam as a metal-free catalyst for CO_2_ was synthesized by chemical vapor deposition (CVD). The 3D hierarchical structure facilitates electrolyte penetration, increasing the interfacial area between graphene foam and electrolyte. The doping of 3D graphene foam with nitrogen heteroatoms provides active sites for CO_2_ reduction at low overpotential, and with high selectivity and durability. This catalyst, synthesized by the chemical vapor deposition (CVD) method, has intrinsic catalytic activity, low onset overpotential (−0.19 V) and a superior activity over Au and Ag, achieving a high Faradaic efficiency of 85% at a lower overpotential (−0.47 V) and better stability for at least 5 h for the formation of CO. A pyridinic N structure has been proposed for N-doped 3D graphene foam as the most active site for CO_2_RR to CO, as shown in Figure 10 [136].

### 4.2. Metal-Free 3D Graphene Nanocompositions (MF3DC)

Low content of N in N-doped graphene-based catalysts limits the activity of CO_2_RR. Nitrogen doping of graphene (N-G) leads to the polarization of carbon due to higher electronegativity of N (3.04) in comparison with that of C (2.55). N-G has four bond configurations, one n-type (graphitic N) and three p-type (edge pyrolytic N, pyridinic N, and nitrilic N) [225]. Pyridinic N changes the electronic structure of carbon atoms and provides adsorption of CO_2_ with the following CO_2_RR to CO [136,225] and formic acid, with an *FE* of 73% at E = −1.84 V (RHE) [226]. In contrast to graphitic N, pyridinic N also facilitates CO_2_RR to C2 an C3 compounds such as C_2_H_4_, C_2_H_6_, C_2_H_5_OH [137].

**Figure 11:**
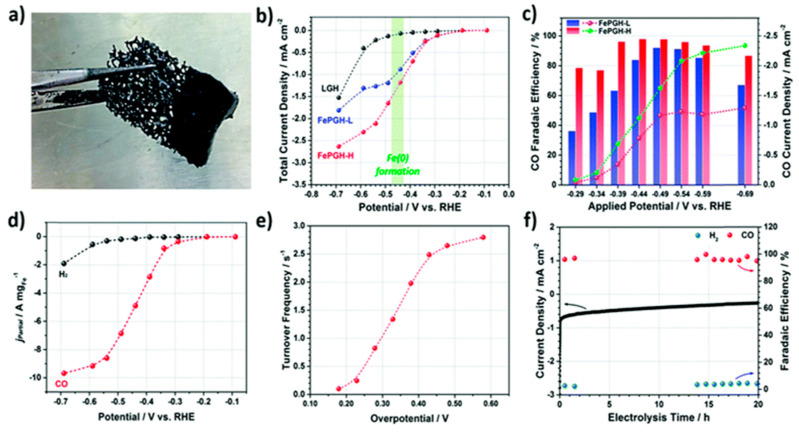
(**a**) Photograph of the prepared FePGH/RVC electrode. (**b**) Total current densities of LGH (black), FePGH-L (blue) and FePGH-H (red) at different applied potentials with Fe(0) formation highlighted. (**c**) CO faradaic efficiencies (bar graph) and CO partial current densities (line graph) obtained by FePGH-L (blue bar and magenta line) and FePGH-H (red bar and green line). (**d**) Mass current densities of H_2_ (black) and CO (red) obtained by FePGH-H electrolysis at different applied potentials. (**e**) Turnover frequencies of FePGH-H at different overpotentials. (**f**) Long-term stability at −0.39 V with respect to current density (black dots) and faradaic efficiency (colored dots) of CO_2_ reduction electrocatalysis by FePGH-H [227]. Reproduced with permission from RSC, 2019.

The deposition of compounds with higher N content, e.g., iron porphyrin on 3D graphene-based materials, such as 3D graphene hydrogel, facilitates the diffusion of electrolyte and electrons. For example, metal-free 3D graphene nanocompositions (MF3DC) have shown synergetic effects from the combination of advantages of 3D graphene (high surface area and conductivity) and metal-free catalysts (stability, low cost). A porphyrin/graphene hydrogel (FePGH) as the catalyst for CO_2_RR into CO exhibits a high Faradaic efficiency of 96.2% at a low overpotential of 280 mV and superior long-term durability for 20 h at −0.39 V (RHE) and i = 0.42 mA cm^−2^ (Figure 11). A reduced LCGO graphene hydrogel (LGH) and FePGH were prepared by hydrothermal treatment with ascorbic acid in a Teflon-lined stainless steel autoclave at 90 °C for 3 h [227].

### 4.3. Non-Noble Metal 3D Graphene-Based Nanocomposite (NNM3DGC)

NNM3DGCs includes deposited non-noble metals or their oxides on 3D graphene networks (3DGN), which increases the interface contact and suppresses the graphene agglomeration, leading to improved electrochemical stability.

MnO deposited on 3D N doped graphene aerogels (NGA) has shown a negligible onset potential of −0.27 V (RHE) and high *FE* of 86% at the E = −0.82 V (RHE), and stability of over 10 h for CO_2_RR to CO. The advanced performance is attributed to the synergetic effect of the high specific surface area of the 3D crumped porous NGA nanostructure and well-crystalized MnO active sites and the suppression of side hydrogen evolution reaction by the doped nitrogen. The catalyst was synthesized by hydrothermal treatment in aqueous solution of GO and hydroxypyridine to form a graphene-based 3D hydrogel, which was immersed in a manganese chloride solution and freeze-dried to disperse and grow nanoparticles (NP) through hydrogel and the incorporation of nitrogen into the graphene lattice. The catalyst MnO/NGA shows the highest content of pyridinic N (38%), in comparison to pyrollic N (35%) and graphitic N (27%), which is important for CO_2_RR [136,225,226]. Pyridinic N sites are anchoring sites for MnO and stimulate its growth. Both pyrrolic and graphitic N strengthen the 3D macroporous graphene aerogel. The morphology of MnO/NGA catalyst (specific surface area 248 m^2^/g) shows an interplanar spacing of 0.223 nm [228].

Additionally, a supported Ni catalyst (Ni-N-MEGO) on porous 3D microwave-exfoliated graphene oxide (3D-MEGO) for CO_2_RR into CO showed a high Faraday efficiency of 72.5%, 78.4%, 89.0% and 92.1% at −0.3, −0.4, −0.55 and −0.7 V, respectively—significantly higher than those of NiPc-G and N-MEGO, with *FE* values of only around 40–71%. The stability of Ni-N-MEGO for CO_2_RR was tested at −0.55 V, and i = 19.3 mA cm^−2^ decreased by 29.1% to ~13.7 mA cm^−2^ after polarization for 21 h. The performance of the catalyst Ni-N-MEGO (onset overpotential of 0.18 V, i = 53.6 mA mg^−1^ at overpotential of 0.59 V) is based on the stabilization of Ni atoms by the coordination with nitrogen (nanopores of <6 nm). Density functional theory (DFT) calculations propose that this CO_2_RR occurs on the edge-anchored unsaturated nitrogen coordinated Ni single atoms, which leads to the enhancement of its rate [229].

### 4.4. Noble Metal 3D Graphene-Based Nanocomposite (NNM3DGC)

Noble metal 3D graphene-based nanocomposites (NNM3DGC) remain the most reliable catalysts among graphene-based catalysts despite the development of alternative metal-free and non-noble 3D graphene-based electrodes for CO_2_RR.

The Pd/three-dimensional graphene-based catalyst (Pd/3D-rGO), In/3D-rGO and Pd-In/3D-rGO) for CO_2_RR to formate were prepared by chemical and hydrothermal methods. The most optimal nanocatalyst, Pd_0.5_-In_0.5_/3D-rGO, with a particle size of 12.8 nm, demonstrates a high Faradaic efficiency of 85.3% at 1.6 V (Ag/AgCl) and a peak potential of 0.70 V (Ag/AgCl), which is more positive than In1.0/3D-RGO (−0.73 V) and Pd1.0/3D-RGO (−1.2 V) [106].

The nanostructured Pt/3D graphene aerogel-based catalyst directly deposited in Cu foam (Pt/GA/CF) 3D binder was developed for CO_2_RR into liquid chemicals (formic acid, acetic acid, propionic acid, methanol, and ethanol) in a TiO_2_ photo anode-driven photo-electrochemical cell. Pt/GA/CF has a better electron transfer capacity than the electrode that combines Pt-modified reduced graphene oxide with CF through polymer binders (Pt/RGO/CF). The uniform dispersion of 3D nanoporous Pt/GA in CF scaffold prevents its self-agglomeration and increases the electrochemical adsorption surface area to 15 times higher than that of Pt/RGO/CF. The carbon atom conversion rate of CO_2_ reduction on Pt/GA/CF markedly increased to 5040 nmol/(h cm^2^) [230].

The three-dimensional graphene-based nanomaterials, either metal-free or as a composite with metals in macroscopic 3D porous interconnected networks, demonstrate high electrocatalytic activity (*FE* up to 86–96% for CO_2_RR to CO), selectivity, and durability for CO_2_RR due to their stable high specific surface area of 3D porous structures, combined with the great mechanical strength and conductivity of graphene, as well as fast mass and electronic transport.

## 5. Porous Materials-Supported Nanocatalysts as 3D Cathodes

**Figure 12:**
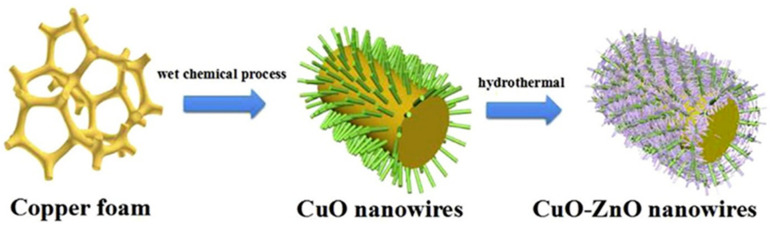
Process for the production of CuO–ZnO nanowires on copper foam [239]. Reproduced with permission from Elsevier, 2019.

3D porous structured electrodes are highly desirable in energy conversion and storage applications, since they not only possess large surface areas to increase the number of active sites and suppress the aggregation of active sites through anchoring effects, but also decrease the contact resistance and hence facilitate electron transfer. In addition, multidimensional pores can maximize fluid flow toward and out of the core of the electrode. Hierarchically porous materials with multiple porosities over lengths in the micro, meso and macro ranges have been intensively used as catalysts and catalyst supports [231,232,233,234] in various applications, including energy storage and conversion fields [235,236,237]. The synthesis techniques used to prepare hierarchically porous materials have been thoroughly reviewed in Yang’s paper [238], where they divided the methods into four types, including (1) basic technologies involving surfactant templating, replication, sol–gel controlling and post-treatment), (2) chemical technologies such as emulsion templating, phase separation, zeolitization and self-formation, (3) replication technologies based on colloidal crystal templating, bioinspiring process and macroporous polymer templating, and (4) physical–chemical technologies including supercritical fluids, freeze-drying, breath figures, and selective leaching. The processes, advantages, features and mechanisms of each synthesis strategy were discussed in detail in Yang’s paper, and will not be presented in this paper. Figure 12 shows a 3D porous Cu foam electrode with hierarchical ZnO nanowires grown on CuO nanowires through combination of chemical and hydrothermal processes [239]. One of the objectives of this paper is to demonstrate the applications of porous materials-supported nano-catalysts as 3D cathodes for CO_2_RR, i.e., a porous structure and its fabrication method enhancing the performance of CO_2_RR.

For practical application of CO_2_RR, current density should be above 200 mA/cm^2^, Faradaic efficiency above 90%, and stability more than 1000 h at a potential more positive than −0.6 V [12,240]. However, the performance of known catalysts is still far from these targets. Therefore, an efficient CO_2_ reduction catalyst should be engineered to have both highly exposed active sites and fast electron and mass transport due to poor electron/mass transportation in the CO_2_ reduction process. Particularly, control and optimization of electrode structure and morphology with tunable pore structure, size, volume and surface area are crucial for the potential enhancement of the accessibility of reaction active sites and mass transfer of the reactants, intermediates and products. Recently, porous substrate supported catalysts have been demonstrated for CO_2_ reduction from macro structure of support to nano-engineering of catalyst as 3D cathode.

### 5.1. D Cathode with Metal-Based Catalysts

A new type of flow-through membrane reactor based on a hierarchically ordered platinum nano-channel array (HPNA) with macrospore channels in combination with mesoporous walls has been developed for CO_2_ conversion applications [241]. The HPNAs were prepared through a dual-templating approach using reverse, porous poly(methyl methacrylate) as a hard template and non-ionic surfactant octaethylene glycol monohexadecyl ether as a lyotropic liquid crystal (LLC) precursor. The HPNA possesses a uniform, hexagonally arranged array of macropores with a thickness and pore diameter of about 10 μm and 250 nm, respectively, and uniformly distributed mesopores with a porosity of 32.5% and pore size of ~2−3 nm. A large number of single-crystalline domains with (110) facets were observed on the mesoporous Pt surface despite the polycrystalline nature of the HPNA. This fabrication method can be easily scaled up which may open a new avenue toward the practical use of CO_2_ for large-scale production of liquid fuels. This new type of flow-through membrane reactor based on a HPNA exhibits high activity and selectivity in CO_2_ conversion producing methanol and ethanol as the dominant liquid products. The Faradaic efficiency and yield for alcohol production are up to 23.9% and 2.1 × 10^−8^ mol s^−1^ cm^−2^ at 51 mA cm^−2^, respectively. No obvious decay was observed in the current density at −2.05 V (vs. RHE) over 10 h of testing. The superior performance results from the three-dimensional architecture, in which the macro-channel arrays enable efficient mass transfer to and from the interfaces while the mesoporous walls provide a large number of highly catalytic active sites for CO_2_ adsorption and reduction.

A dealloying process has been reported in a few alloyed systems [77,242], and the resulting materials have shown unique catalytic performance such as in fuel cells [243,244] and alcohol oxidation [245]. However, there are few reports on their catalytic properties for CO_2_ reduction. Lu et al. [246] reported a dealloying method to prepare a porous Ag catalyst for CO_2_RR. The Ag catalyst was obtained by two-step dealloying of an Ag–Al precursor using an aqueous HCl solution. By selectively etching Al through dealloying, the remaining Ag atoms were reorganized to form a 3D interconnected nanoporous structure. This method can not only create an extremely large surface area for catalytic reaction (ca. 150 times greater than polycrystalline silver), but also the curved internal surface generates a large number of highly active step sites for CO_2_ conversion (at least 20 times more active than polycrystalline silver), resulting in exceptional activity that is over three orders of magnitude higher than that of the polycrystalline counterpart at a moderate overpotential of <500 mV. K. More importantly, this CO_2_ electroreduction activity has been achieved with a CO Faradaic efficiency of 92%.

A facile one-step electrodeposition technique by using hydrogen bubbles as a dynamic template was reported by Ye et al. [247] for the fabrication of 3D hierarchical SnOx@Sn-Cu/Sn core–shell catalysts supported on Cu foam. They demonstrated that the SnO_x_ shell with a hierarchical Sn–Cu/Sn core can be reconstructed in situ under cathodic potentials of CO_2_RR. The hierarchically heterogeneous Sn–Cu alloy/Sn metallic core responsible for high electrical conductivity and amorphous thin SnO_x_ shell for catalyzing CO_2_RR. In situ reconstruction of a 3D hierarchical Sn–Cu/SnO_x_ core/shell catalyst achieves a high current density of 406.7 +/− 14.4 mA cm^−2^ with C1 Faradaic efficiency of 98.0+/−0.9% at −0.70 V vs. RHE, and remains stable at 243.1 +/− 19.2 mA cm^−2^ with a C1 Faradaic efficiency of 99.0 +/−0.5% for 40 h at −0.55 V vs. RHE. Under the cathodic potentials of CO_2_RR, the SnO_x_ shell was reconstructed in situ with that the hierarchical Sn-Cu alloy/Sn core provides sufficient Sn sources for Sn diffusion into the shell and trigger the shell reconstruction, and the surface structure and composition of catalytic shell can be accurately tuned by the devisable bulk composition.

Li et al. [200] prepared mesoporous SnO_2_ nanosheets on carbon cloth (SnO_2_/CC) by a hydrothermal method. Firstly, a strip of CC was immersed in an autoclave containing SnCl_4_ and thioacetamide dissolved in isopropanol for hydrothermal growth of SnS_2_ nanosheets on CC (SnS_2_/CC). Secondly, the SnS_2_ precursor was converted to mesoporous SnO_2_ with retained nanosheet morphology via a simple calcination process in the air atmosphere. In principle, this synthetic procedure is also applicable to larger-sized electrodes simply by increasing the size of the autoclave and also allows growth of catalysts on various other stable electrodes. SnO_2_/CC electrode can also be directly used as the electrode for CO_2_RR without substrates or binders. The 3D hierarchical porous SnO_2_/CC electrode exhibits a partial current density of about 45 mA cm^−2^ at a moderate overpotential (−0.88 V) with high Faradaic efficiency (87 +/− 2%). The overpotential needed to reach the maximum *FE* for formate is also lower than most Sn or heteroatom-doped carbon-based electrodes. Zhang et al. [197] reported a hydrothermal method for the synthesis of high surface area SnO_2_ nanoparticles on high surface area carbon supports (carbon black and graphene). The formed 3D porous structures facilitate CO_2_ transport and reduction. In particular, the efficiency reached a maximum faradaic efficiency for formate production of >93% on 5 nm of SnO_2_ particles with current densities of over 10 mA cm^−2^ on high surface area graphene supports. These catalysts are very stable during electrolysis and can continue producing formate for at least 18 h.

### 5.2. D Cathode with Carbon-Based Catalysts

One of the traditional electrode materials used as a porous, three-dimensional electrode is carbon foam, particularly in the 97% vol. porous form of reticulated vitreous carbon (RVC) [236,237]. An advantage of this open structure, in contrast to other carbon foams, is that flow permeability is high, which can offer moderate mass transport rates. However, RVC exposes a flat surface not likely to enhance electron transfer, which limits RVC as an electrode material for electrochemical reactions. Flexer et al. [105] synthesized a NanoWeb–RVC material by a modified chemical vapor deposition (CVD) strategy, allowing for the direct growth of carbon nanotubes (CNTs) on RVC [248]. NanoWeb appears as a fine fur or roughness on the surface, distinct from the characteristic flatness of RVC. The synthesis of NanoWeb–RVC was achieved by coating over RVC Fe(III) para-toluenesulfonate in ethanol, which acted as a catalyst. In a thermal CVD system at 600 °C, H_2_ reduced Fe(III) to form iron nanoparticles, and then the growth of the carbon nanotube layer was initiated at 800 °C by flushing the system with Ar/acetylene/H_2_. Electrophoretic deposition (EPD) is a well-established methodology for generating film on scale-up electrodes of a few cubic centimeters in size [249,250]. In the preparation of EPD-3D, CNTs’ synthesis and their deposition on the RVC are two separate steps. RVC was placed in a beaker and connected to the positive terminal of the power source, with a stainless-steel mesh cylinder as a cathode, i.e., a two-electrode setup, amenable to scale-up. EPD occurred in two steps, initially electrophoresis, wherein the negatively charged CNTs migrate to the positively polarized RVC, followed by deposition, due to coagulation. Since CNTs can be synthesized through a large variety of techniques, and therefore CNTs of any diameter, can be deposited on RVC. The NanoWeb–RVC electrode showed an impressive performance as a microbial cathode compared to RVC and graphite plate electrodes, having only been surpassed by EPD-3D, which, with an identical macrostructure and a closely related nanostructure, can be considered an upgraded version of NanoWeb–RVC. The outstanding performance of these novel biocathodes, with current densities up to −200 A m^−2^ and acetate production rates up to 1330 g m^−2^ day^−1^, and with electron and CO_2_ recoveries into acetate being around 100%, are among the highest reported by either designed or commercially available biocathodes.

The construction of hierarchically structured nano-porous N-doped carbon/carbon nanotube composite membrane (HNCM/CNT) was prepared via a bottom-up method by Wang et al. [193]. A homogeneous multi-wall CNTs dispersion was firstly prepared by sonicating CNTs in a solution of poly [1-cyanomethyl-3-vinylimidazolium bis(trifluoromethanesulfonyl)imide] (PCMVImTf2N) and poly(acrylic acid) (PAA) indimethyl formamide (DMF). The stable polymer/CNTs dispersion was then cast onto a glass plate, dried at 80 °C and finally immersed in an aqueous NH_3_ solution to build up the nano-porous polymer/CNTs film membrane. Afterwards, pyrolysis of the porous polymer/CNTs film membrane at 900 °C in N_2_ leads to the targeted HNCM/CNT membrane. The fabrication method is straightforward and easy to adapt to larger-sized membrane fabrication, and their properties can be optimized using a range of polymeric ionic liquids and carbon nanostructures. The membrane is demonstrated to function as a binder-free, high-performance gas diffusion electrode for the electrocatalytic reduction of CO_2_ to formate. The Faradaic efficiency for the production of formate is 81%, and the overpotential required to reach the maximum Faradaic efficiency is −0.9 V vs. RHE, with an excellent long-term stability of 36 h. The hierarchical porous architecture of this membrane can act as a standalone electrode without the need for a binder. In such porous N-doped carbon, the macropores provide mass transport highways while the mesopores and micropores provide a large surface area and high population of spatially accessible electroactive sites for the enhancement of the three-phase contact and charge transport between the electro-catalyst, aqueous electrolyte, and gaseous CO_2_ reactant. Furthermore, the nitrogen species incorporated into the carbon framework improve the electrochemical stability and most importantly constitute the active sites for CO_2_RR.

Ni et al. [251] recently developed F-doped porous carbon with a cage-like morphology as an efficient catalyst for CO_2_RR. The current density and Faradaic efficiency for CO product exhibits a dramatic increase through engineering mesopores/micropores structure at the surface of F-doped carbon shell. The optimized F-doped cagelike carbon (F-CPC) possesses a large surface area with moderate mesopore and abundant micropores, as well as high electrical conductivity, and exhibits Faradaic efficiency of 88.3% for CO at −1.0 V vs. RHE with a current density of 37.5 mA·cm^−2^. Moreover, F-CPC retained a steady Faradaic efficiency for the CO product throughout 12 h durability tests. The synthesis procedures of F-CPC samples follow an aldol reaction where SiO_2_ particles were used as templates. An aldol reaction was conducted at the surface of SiO_2_ by using resorcinol and formaldehyde as precursors. Polytetrafluoroethylene (PTFE) was simultaneously introduced as the fluorine source during the aldol reaction. After calcination at 900 °C, the obtained sample was further treated by CO_2_ activation to create open pores on the carbon shell. Finally, the SiO_2_ templates were removed by etching with HF.

**Figure 13:**
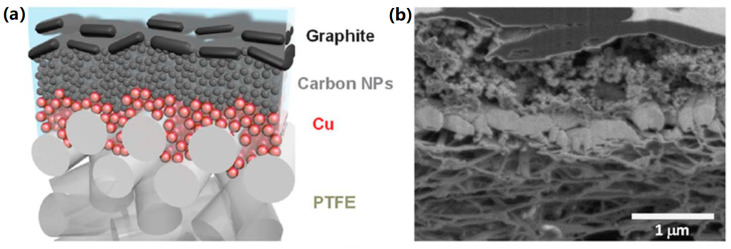
Structure and performance of the polymer-based gas diffusion electrode. (**a**) Schematic illustration of the graphite/carbon NPs/Cu/PTFE electrode; (**b**) Cross-sectional SEM image of a fabricated graphite/carbon NPs/Cu/PTFE electrode [96]. Reproduced with permission from Science, 2018.

Hydrophobic polymers have been used as support to improve the conventional carbon-based electrodes to prevent loss of hydrophobicity and flooding [96]. For example, C. Dinh et al. sputtered Cu catalysts onto a porous PTFE support and then coatings of nanoparticles and graphite were applied on the top as shown in Figure 13. The carbon nanoparticles and graphite coatings provide the electrical conducting paths since PTFE is a highly insulating but hydrophobic material. During a test in 7 m KOH for 150 h at an applied cell potential of −0.55 V vs. RHE, the current density gradually decreased from 100 to 75 mA cm^−2^ and the *FE* for ethylene remained at 70%. The PTFE-supported cathode produced a 300-fold increase in the operating lifetime of the electrode when compared to carbon-supported electrodes, which lost hydrophobicity within one hour. The low over-potential and high selectivity have been primarily attributed to hydrophobicity improvement of the cathode structure with a gas-diffusion layer.

### 5.3. D Cathode with MOFs/Single-Atom Metal Catalysts

Metal–organic frameworks (MOFs), as an attractive precursor, could be used to fabricate hybrid catalysts containing metal (oxides) nanoparticles, porous carbon materials and doped porous carbons as a promising candidate for CO_2_ electroreduction to maximize the use of single-atom metal sites. For example, Li et al. [252] recently reported Cu MOF-derived Cu_2_O/Cu anchored in N-doped hollow porous carbon (Cu_2_O/Cu@NC) for increasing the selectivity and activity of electrochemical CO_2_-to-formate conversion. In Li’s work, 1,3,5-benzenetricarboxylic acid (H3BTC) provided the precursor of the carbon framework. At the same time, nitrogen derived from benzimidazole was successfully doped in situ into the carbon matrix. Porous Cu_2_O/Cu@NC materials were prepared via carbonization of the obtained Cu MOF precursors at different temperatures (700, 800, and 900 °C) for 5 h in an inert atmosphere. When carbonized at 800 °C, the prepared catalyst (Cu_2_O/Cu@NC-800) exhibited a remarkable improvement in activity and selectivity toward formate and maximum faradic efficiencies for formate reached 70.5% at −0.68 V. The long-term stability of Cu_2_O/Cu@NC-800 was maintained for a continuous 30 h of electroreduction at −0.68 V with a current density of 4.4 mA cm^−2^ and Faradaic efficiency around 66.5%.

Zhao et al. [253] reported an ionic exchange of MOF to access single Nickel sites for efficient electroreduction of CO_2_ to CO. The synthesis is based on ionic exchange between Zn nodes and adsorbed Ni ions within the cavities of the MOF. The authors developed a ZIF assisted strategy to generate Ni single atoms distributed in nitrogen-doped porous carbon (Ni SAs/N-C) for active CO_2_ reduction. The ZIF-8 was first dispersed in n-hexane, followed by the injection of Ni(NO_3_)_2_ aqueous solution. Through a double-solvent approach, the Ni precursor could be confined within the pores of ZIF-8. The mixture was pyrolyzed at 1000 °C in Ar, during which the organic linkers were transformed into a N-doped carbon skeleton and Zn nodes evaporate leaving the N-rich defects. These sites would be easily occupied by the neighboring Ni^2+^ ions, who can be stabilized by N coordination and further reduced by the surrounding carbon to single Ni atoms. This single-atom catalyst exhibited an excellent turnover frequency for electroreduction of CO_2_ (5273 h^−1^), with a Faradaic efficiency for CO production of over 71.9% and a current density of 10.48 mA cm^−2^ at an overpotential of −0.89 V. Ni SAs/N-C exhibited a long-term stability of 60 h of operation, during which no obvious decay in Faradaic efficiency and current density was detected.

To maximize the use of single-atom metal sites, H. Yang et al. [254] developed a strategy by constructing a free-standing, cross-linked and high-yield carbon membrane (denoted as CoSA/HCNFs) by combining MOF material into electrospinning process. Firstly, the primary fibers were obtained from the mixture solution of ZIF-8 nanoparticles, Co(NO_3_)_2_ and polyacrylonitrile (PAN) in DMF via electrospinning technique. The as-spun nanofibrous materials were pre-oxidated in air at 220 °C for 1 h and then carbonized under Ar gas flow at an optimal temperature of 900 °C for 1 h to achieve a high graphitization. Then, the resultant materials were washed thoroughly in a H_2_SO_4_ solution for 12 h to remove residual Zn species. The nitrogen component and reticulated structure of CoSA/HCNFs were derived from the decomposition and collapse of PAN, ZIF-8 nanoparticles and Zn species. Co ions were reduced by carbonized organic polymers and anchored by N-doped carbon. The polymer film was obtained after carbonization and etching. The CoSA/HCNFs sample had a diameter of approximately 500 nm and a desired cellular structure with an average size of ca. 70–100 nm. Both mesopores and macropores can be found on CoSA/CNFs nanofibers using the BET method. The method for preparation of single-atom and self-supporting CoSA/HCNFs membranes exhibits >90% Faradaic efficiency along with >200 mA cm^−2^ current densities for CO production, which is one of the best results ever reported by single-atom catalysts so far. The obtained hierarchically porous, crosslinking and free-standing carbon structures generate large electrochemical active surface and abundant channels for electron and reactant transportation, leading to a much higher density of effective Co single atoms as active sites for CO_2_RR. This strategy might represent a very important step forward to the scalable synthesis of efficient single-atom catalysts and industrial application of CO_2_ electroreduction.

The importance of the porous structure has been demonstrated in catalyst/electrode materials for CO_2_RR. By tailoring the pore size of porous materials from micropores to macropores, the performance of CO_2_RR can be improved. Macropores (>50 nm) provide facile transfer and diffusion of reactants and products in the reactions over the electrodes, mesopores (2–50 nm) and micropores (<2 nm) can provide a high surface area to form more active sites with high dispersion. Thus, hierarchical porous materials within integrated properties are desirable for accelerating mass transport and improving CO_2_ conversion efficiency.

Despite the progress, it seems that porous nano-CO_2_RR electrocatalysts are still far from reaching the technical requirements for commercialization. Fundamental understanding of the structure-property relation is a pre-requisite for the development of highly efficient hierarchical porous structure electrocatalysts. Various in situ diagnosis tools should be used to directly investigate the reduction reactions and help identify optimal synthesis strategies for controlling the structure and components of catalysts. Furthermore, the integrated information from the experimental results and diagnosis tools with 30 ultiphysics modeling may bring deeper understanding on the relationship of transport phenomena, fluid flow, and electrochemical reactions within electrodes with hierarchically porous structure, which would facilitate the rational design of high-performance catalysts as well as the prediction of the structure–performance relationship.

## 6. Modeling and Simulation

### 6.1. Modeling Electrochemical CO_2_ Reduction

Despite the complex nature of the electrochemical CO_2_ reduction reaction (CO_2_RR) that pertains to the mechanisms and pathways that determine the catalytic activity and selectivity, significant advances have been made recently in the design and development of improved electrocatalyst materials [66]. From a cell or device level perspective, high catalytic performance relies on a well-balanced interplay of cell configuration, electrode composition and micro-structure, local pH at the catalyst, type of electrolyte, and external conditions, such as environmental pressure and temperature, during cell operation [255,256]. To somewhat alleviate the complexity in electro-kinetics models of the CO_2_RR, CO and HCOO^−^ are often considered as product species [257,258], which simplifies the kinetics due to the two-electron character of the overall reaction thus involved. The latter causes, however, significant limitations to the applicability of those kinetic models, especially for cell design and catalyst scale-up.

First principles calculations and electro-kinetic modeling are highly insightful for efforts to decipher the CO_2_RR and identify the role of key intermediate species and elementary steps [255,259]. The microkinetic models usually ignore mass transport processes at the electrode level, thus assuming uniform reaction conditions [259,260]. However, it has been demonstrated that forays in materials and cell design are more likely to succeed if microkinetic modeling of reaction mechanisms and pathways at the electrocatalyst surface is combined with the macroscale modeling of mass transfer processes at component and cell levels [99,260]. Mass transport effects may largely control the net rate of the CO_2_RR, in particular for nanostructured porous electrodes. Although the majority of models that are applied to mass transport in CO_2_RR system are limited to planar electrodes, there have been recent attempts to advance those models to non-planar electrodes such as nanowire and porous electrodes [99,115,257].

The crucial metrics for CO_2_ reduction processes are Faradaic efficiency, current density, and energy efficiency [255]. The Faradaic efficiency is defined as the ratio of the amount of charge consumed to produce a certain product to the total charge supplied to the system (*Q*),

(1) $$FE_{product}=\frac{yNF}{Q}.$$

Here, *y* is the number of moles of product formed and *n* is the number of electrons transferred in the half-cell reaction [261]. Energy efficiency (*EE*) is defined as the ratio of the net energy that is used for producing a specific product [255],

(2) $$EE_{product}=\frac{E^{0\;}\;\times \;FE_{product}}{E^{0\;}+\eta },$$

where *E*° is the equilibrium cell potential and *η* is the total overpotential.

The type of catalyst material plays an important role in determining catalytic properties, elementary steps, and the overall mechanism and pathway of the reaction. Earlier mechanistic studies have focused on obtaining key catalytic parameters related to performance, total yield, selectivity, and Faradaic efficiency under hard assumptions such as absence of mass transport limitation, low intermediate coverage and steady-state reaction conditions [260,262,263].

In this section, we review the recent advances in CO_2_RR micro-kinetic and mass transport modeling studies. We revisit the assumptions and limitations underlying different modeling approaches and conclude the section by delving into future perspectives and recommendations.

#### 6.1.1. Intermediates and Reaction Pathway

Tafel slopes are widely used as composite metrics to assess and compare the activity of catalytic materials and electrodes. Changes in Tafel slope over a scanned range of electrode potential indicate that different elementary reaction steps and different reaction intermediates control the overall rate of the reaction; a particular value of the Tafel slope may thus be identified with a particular rate determining step or rate determining term in the multistep mechanism [52,116,264]. Moreover, the doubling of the Tafel slope could be indicative of the transition from a kinetically limited regime at low electrode overpotential to a mass transport-limited regime at high overpotential [255,261].

For the CO_2_RR, the formation of bicarbonates in producing various reaction intermediates such as COOH* have also been the subject of other recent studies [116,255,261]. Proton transport and water are also involved in producing CO on Ag electrode. Calculations using DFT and electro-kinetic models, supported by experimental studies, have been invoked in finding mechanistic interpretations of transitions in Tafel slopes [52,92,255]. CO_2_RR activities of various metal electrodes have been studied with DFT-based computation [255,256]. These studies relate the electro-catalytic activity to the binding energies of chemisorbed reaction intermediates. Nørskov and co-workers, for example, suggested that CO is produced through adsorbed COOH* intermediate [265]. This was further confirmed by experimental data, where the effect of the intermediate binding energy on electrocatalytic properties was studied in detail [92]. DFT studies also established a correlation between intermediate binding energies and catalyst reactivity during electrochemical conversion of CO_2_ to CO or HCOOH [265]. The volcano plot of CO partial current density was introduced with respect to COOH* and CO* binding energies [264].

As one of the most selective catalysts for CO_2_RR, the electrocatalytic properties of Au have been extensively studied in aqueous conditions, in particular using electrochemical cells such as rotating ring disk electrodes (RRDE) [48]. The hydrogen evolution reaction (HER) is an important competing process to the CO_2_ reduction to CO. RRDE has been employed to study the role of mass transport limitations among these two competing reactions on the Au surface [266]. Goyal et al. [257] observed that changes in the local pH caused by mass transport effects can considerably influence the selectivity of the Au catalyst toward formation of CO. The increase in selectivity is mainly attributed to the inhibition of the competing HER reaction under a high mass transport regime. Overall, enhancement in selectivity in this case is a direct result of controlling mass transport.

#### 6.1.2. Product Selectivity

The role of concentrations of reactive species on the selectivity of the Au electrode for CO production has been studied in detail [48,66,257]. As gleaned from those studies, interfacial concentration gradients of reactive species in bicarbonate electrolytes such as CO_2_, HCO^3−^, OH^−^, and H+ control the competition between CO_2_RR and HER. Rates of transport of CO_2_ from the bulk to the interface and of OH^−^ ions from the interface to the bulk are highly sensitive to these concentration distributions and thus determine the selectivity [257,266].

Three-dimensional (3D) porous electrodes have also been used for CO_2_RR, where the porosity of the catalytic medium provides an additional parameter to tune concentration gradients of active species and thereby control the selectivity [255,262]. Experimental studies have shown that the selectivity of CO_2_RR can be considerably improved by increasing electrode porosity.

**Figure 14:**
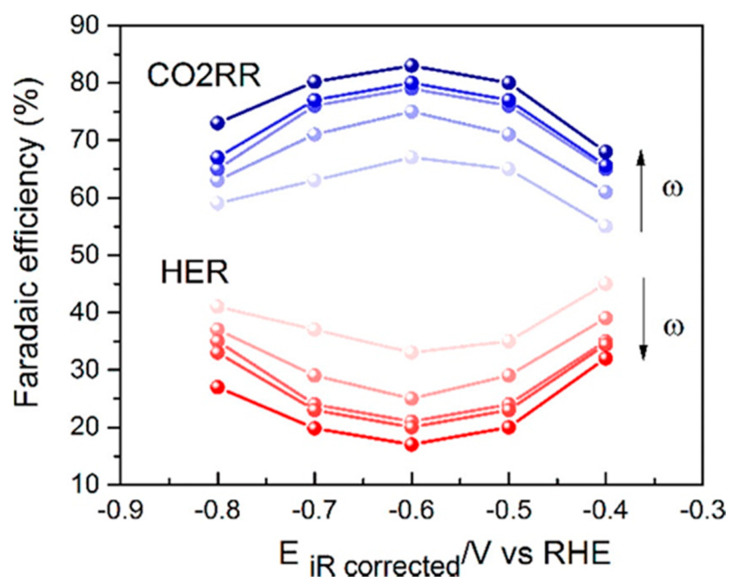
Faradaic efficiency for CO formation and HER reaction vs. electrode potential (vs. RHE) and rotation rate (ω) for the Au polycrystalline surface. The direction of the arrow indicates increasing rotation speed, from 800 to 2500 rpm [257]. Reproduced from an open access article from ACS Publications, 2020.

The porosity of the electrode can reduce the impact of the HER on the selectivity of the CO_2_RR for producing CO [66,260,262]. The selectivity of CO production has been shown to be insensitive to the thickness of the porous Au catalyst [260]. Figure 14 shows the experimental results from rotating disk electrode (RDE) experiments that were performed to reveal correlations between mass transport rates, electrode potential, and CO_2_RR Faradaic selectivity [260]. The same study by Koper and co-workers [257] confirmed that a maximum Faradaic efficiency of 83% for CO formation is achieved at −0.6 V (vs. RHE).

For an RRDE configuration, the *FE* for CO is calculated from,

(3) $$FE_{CO}=\frac{i_{ring}\times 100}{\left|i_{disk}\right|\times N}$$

where *i_disk_* is the total current on the disk during RRDE measurement, *i_ring_* is the experimental ring current, and *N* is the experimentally determined apparent collection efficiency [257]. The latter is quantitative measure of relative contributions due to competing CO_2_RR and HER. Please note that the Faradaic efficiency of the HER can be calculated using

(4) $$FE_{HER}=100-FE_{CO}$$

**Figure 15:**
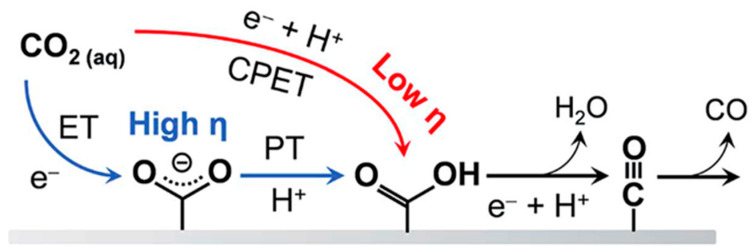
The schematic of CPET reaction mechanism for electroreduction of CO_2_ to CO on a Ag electrode [259]. Reproduced with permission from ACS, 2015.

The variation of the partial current density for the formation of HCOO^−^ as a function of pH at various potentials is provided in Ref. [57,257]. By increasing pH at low overpotentials, the current density attributed to production of HCOO^−^ species increases [262]. A reaction mechanism for the electroreduction of CO_2_ to CO on a Ag electrode based on a concerted proton–electron transfer (CPET) is depicted in Figure 15 [255,259]. The reaction intermediates are identified based on in situ ATR-FTIR spectroscopy, which vary depending upon the potential range [267]. For instance, CO_2_^−^* and COOH* are the main intermediates at highly negative overpotential [255]. Changes of the metal valence state are proposed as the main reason for the dependence of the reaction mechanism to the potential of the metal electrode [268].

To summarize this section, previous studies have shown that a detailed understanding of microkinetic mechanisms and pathways is essential for rationalizing correlations between electrode potential and CO production rate and for determining the Faradaic efficiency and selectivity of the CO_2_RR. Competing processes such as HER can be controlled by inducing required mass transport limitations during CO_2_RR. RRDE are used for de-convoluting the impact of mass transport on competing production rates of H_2_ and CO, while the design of porous catalytic media can yield similar effects at the cell level. Tuning the porosity and pore space morphology of 3D porous electrodes, in particular when exposed to a bicarbonate electrolyte, can be an effective means to control the relative rates of competing HER and CO_2_RR. One should note that other microkinetic indicators such as reaction and activation Gibbs energies of elementary reaction steps and corresponding rate constants, as well as the strength and concentration of various anionic/cationic electrolyte types and the types of CO_2_RR products can have significant influence on the observed trends for the impact of mass transport conditions [99,269]. In the next section, we will review mass transport models that have been employed for 3D porous electrodes during CO_2_RR processes.

### 6.2. Mass Transport Modeling in Nanostructured CO_2_RR Electrocatalysts

Garg et al. recently presented a comprehensive review of CO_2_ reduction processes from modeling and design perspectives [256]. The authors highlighted electrolyzer configuration, electrode structure, pH, type of electrolyte, and operating conditions such as pressure and temperature as the most important factors impacting the efficiency of CO_2_ reduction processes. In particular, mass transport rates of active species, including the transport of CO_2_ dissolved in the electrolyte were referred to as the main factor that may influence the CO_2_ reaction mechanism and pathway as well as the overall rate and selectivity of the process.

The majority of modeling studies of mass transport effects in CO_2_RR have explored planar electrodes [115,116,256]. A planar geometry imposes a higher mass transport limitation on the electrochemical cell than that in a 3D porous electrode, which may hinder the transport rate of active species from bulk of electrolyte to the electrode surface [99,262]. Two major designs are proposed in the literature to minimize the effect of mass transport on performance. In one approach, porous electrodes are made as gas diffusion electrodes (GDE). GDEs can potentially enhance the current density compared to that in a planar electrode design [99]. They, however, introduce added complexity to the operation of cell because of the multi-layer and complex nature of the electrodes. In the second approach, nanostructured porous structures are formed by coating electrocatalyst materials on arrays of carbon nanotubes or similar nanostructured materials such as Cu nanowires [115,262]. Such a design provides an intrinsic control over geometrical factors of the electrocatalytic medium and it may be useful for enhancing the selectivity of the electrocatalysts. Notably, the *FE* in this type of electrocatalyst configuration depends strongly on the total surface coverage of active oxide materials. Together with lower proton concentration in the bulk and the type of reactant species at the interface, the total surface coverage of active species can control the competition between HER and CO_2_RR [115].

Here, we summarize mass transport models for two types of porous electrodes: conventional porous gas diffusion electrode and 3D Cu nanowire electrodes. The key operational parameters, for instance concentration of active species such as CO_2_, HCO_3_, and OH- and local pH, are calculated by solving the coupled system of transport and reaction equations [99,115,116]. These approaches allow exploring and exploiting the importance of mass transport processes for improving selectivity and efficiency of the CO_2_RR processes, in particular in high surface area electrode configurations.

#### 6.2.1. Modeling Mass Transport in a Porous CO_2_RR Gas Diffusion Electrode

**Figure 16:**
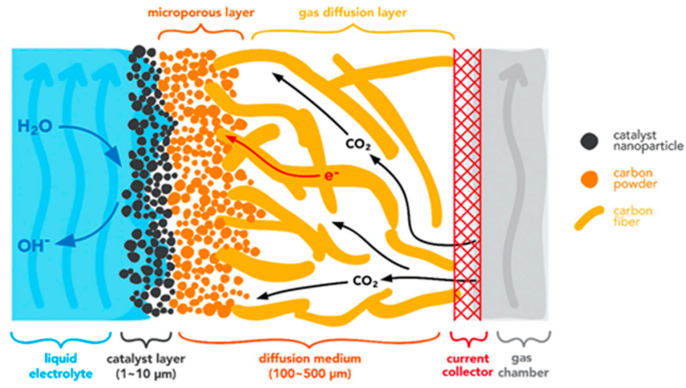
Schematic of a gas diffusion electrode for CO_2_RR [99]. Reproduced with permission from RSC, 2018.

Weng et al. [99] employed a multi-physics approach to investigate the combined impact of micro-kinetics and transport in the performance of a CO_2_RR cell. The model comprises a multi-layer configuration of gas diffusion electrode (GDE) including catalyst layers (CL), electrolyte, porous diffusion medium, gas collector, and current collectors, as depicted in Figure 16.

Depending upon wettability of the catalyst layer and its porosity, various diffusion regimes could occur at the interface between catalyst layer and diffusion medium. As illustrated in the following reactions, CO_2_ directly dissolves in the electrolyte, while other species such as bicarbonates produce other intermediates [99].

(5) $${\mathrm{CO}}_{2}\left(\mathrm{aq}\right)+H_{2}O\overset{k_{1}k_{-1}}{↔}H^{+}+{\mathrm{HCO}}_{3}^{-},\;\;K_{1}=10^{-6.37}\;$$

(6) $${\mathrm{HCO}}_{3}^{-}\overset{k_{2,}k_{-2}}{↔}H^{+}+{\mathrm{CO}}_{3}^{2-},\;\;K_{2}=10^{-10.32}M\;\;\;\;$$

(7) $${\mathrm{CO}}_{2}\left(\mathrm{aq}\right)+{\mathrm{OH}}^{-}\overset{k_{3,}k_{-3}}{↔}{\mathrm{HCO}}_{3}^{-},\;\;K_{3}=K_{1}/K_{w}\;\;\;$$

(8) $${\mathrm{HCO}}_{3}^{-}+{\mathrm{OH}}^{-}\overset{k_{4,}k_{-4}}{↔}H_{2}O+{\mathrm{CO}}_{3}^{2-},\;\;K_{4}=K_{2}/K_{w}\;$$

(9) $$H_{2}O\overset{k_{w,}k_{-w}}{↔}H^{+}+{\mathrm{OH}}^{-},\;\;K_{w}=10^{-14}M^{2}\;\;\;\;\;$$

The primary governing equations for calculating equilibrium concentration are based on continuity and transport equations [256]. The transport equations of gaseous species consist of diffusive terms and convective terms. Effective diffusivities can be estimated using the Stefan–Maxwell equation that accounts for Knudsen and molecular diffusion [99]. This baseline model is able to predict that by increasing the active surface area of a GDE and lowering the mass transfer resistance, the CO_2_RR performance increases significantly. Weng et al. [99] showed that a reduction in catalyst loading (i.e., the Pt mass ratio per geometric area) by a factor of two in the regime of low overpotential, causes a reduction in the CO current density by the same factor of two. This behavior is indicative of a kinetically controlled regime with negligible contribution of mass transport effects. The proportionality between Pt loading and current density wanes at higher overpotential until the trend reverses beyond 1 V vs. RHE. As expected, at high current densities, CL becomes flooded and a lower catalyst loading at the flooding regime can actually enhance the CO partial current density. Mass transport rate and gas permeabilities of active gas species can be improved by increasing the porosity, which can result in higher effective diffusivities in a wetted vs. a flooded CL [256].

The contact angle of the CL was also shown to have a significant effect on current density and *FE* for CO production. Both CO current density and *FE* decrease after reaching a maximum level [262]. Overall, higher consumption of CO_2_ will lower CO current density and *FE*, which is generally the case for a planar electrode vs. 3D porous electrodes. Such insights gained from the mass transport model in Ref. [262] are highly important for the design and fabrication of highly efficient GDEs for CO_2_RR.

#### 6.2.2. Effect of Mass Transport of Catalyst Activity of 3D Nanowire Electrodes

**Figure 17:**
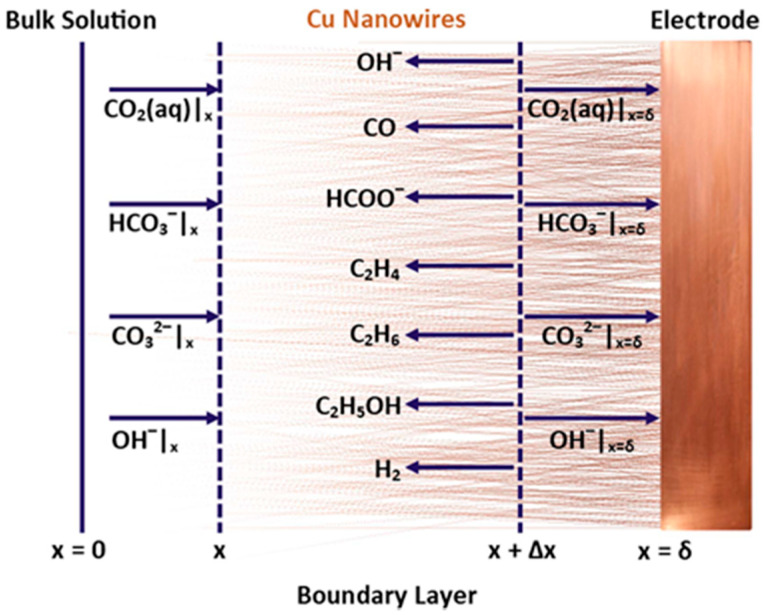
Schematic of the mass transport model for electroreduction of CO_2_ on Cu nanowires [115]. Reproduced with permission from IOP Publishing, 2017.

Raciti et al. [115,116] introduced a mass transport model for the CO_2_RR on Cu nanowire electrodes. A series of partial differential equations (PDE) was developed, where the consumption of various species such as CO_2_ and H_2_ and generation of species such as OH^-^ were used to calculate local concentrations of CO_2_, bicarbonate, OH^-^ and CO_3_^−2^, as illustrated in Figure 17. Solving these PDEs for nanostructured electrodes results in spatial distributions of the electrochemical reactions. The mass transport effect on the electrocatalytic activity and selectivity of the Cu nanowire electrode was then evaluated by accounting for the local pH as a key variable. The correlation among electrocatalytic performance and local pH is an indication of mass transport effects on catalyst activity and selectivity. The model in Ref. [115] clearly supports a specific range of local pH on such nanostructured electrodes, in which the CO_2_RR becomes most effective, in terms of balancing the impact of competing reactions such as HER and improving product selectivity. The mass transport rates are seen to be highly important in such electrocatalytic system with high electrode surface area.

This section provided an overview of typical reaction pathways for CO and HCOO- production, identifying key intermediates and elementary reaction steps with the help of computational studies at the level of density functional theory (DFT) and experimental data. Basic assumptions and methodologies were summarized that have been employed for micro-kinetics and mass transport modeling of the CO_2_RR. In particular, we provided an overview of recent mass transport models that have been applied to electrochemical CO_2_ reduction on copper (Cu) nanowire electrocatalysts and nano-porous electrodes. We also discussed models in this section that describe catalyst layers for CO_2_ reduction cells and their local environments, such as distribution of CO_2_, hydroxyl, and water, which can be greatly affected by operating conditions, catalyst layer properties, and overall mass transport control regimes. Mass transport models are particularly discussed in the context of gas diffusion electrodes for CO_2_ reduction and the interplay between the transport of key species and the overall electrochemical reaction kinetics. The mass transport and further developments of microkinetic models thus provide deeper insights into the complex reaction mechanisms of the CO_2_RR. They identify the importance of electro-kinetic and mas transport models to guide the design and scale-up of advanced CO_2_RR cells.

## 7. Concluding Remarks

### 7.1. Current Status and Achievements

3D cathodes with simultaneously high surface areas and pore volumes are desired to facilitate an increased CO_2_ reduction rate. This work reviewed the current status of the fabrication and evaluation of 3D cathodes based on different nanoscale building blocks. Table 2 summarizes 3D cathodes with different architecture and their related performance for CO_2_ reduction as reviewed in this work. Most of the current densities are very low in this table, since many of these studies employed aqueous h-cell or flow-cell without optimization of conditions. 3D cathodes can be divided into two categories: 3D freestanding cathodes without additional GDLs, and 3D cathodes based on GDL structures. 3D freestanding cathodes avoid potential delamination of layered architecture from GDL structures and potentially increase the long-term durability of the cathode. It has been reported that 3D porous carbonaceous electrodes exhibit efficient electrocatalytic properties for reduction of carbon dioxide into carbon monoxide. A comprehensive review of recent mass transport models that are applied to electrochemical CO_2_ reduction on copper (Cu) nanowire electrocatalysts and nano-porous electrodes was also presented. Models are discussed to describe catalyst layers for CO_2_ reduction cell and their local environment that may be largely affected by operating conditions, catalyst layer properties, and overall mass transport control regimes.

Many studies have concentrated on the deconvolution of mechanistic aspects of the catalysts in the kinetic region and short term performance evaluation. Transport phenomena are particularly critical for self-supported 3D catalysts or electrodes. The three-dimensional graphene-based nanomaterials, either metal-free or composites with metals in macroscopic 3D porous interconnected networks, have demonstrated high electrocatalytic activity (*FE* up to 86–96% for CO_2_RR to CO), selectivity, and durability for CO_2_RR due to the stable high specific surface area of 3D porous structures combined with improved mechanical strength and conductivity of graphene, as well as increased mass and electronic transport.

Nanofibers as building blocks for 3D cathodes construction have been widely studied as a cost-effective method and have shown promising results for increasing mass transport, with macro-porous architectures and large surface areas as sites for CO_2_RR. Particularly, the feasibility of 3D freestanding cathodes has been demonstrated successfully without the need for additional GDLs. Nanofibers as building blocks for 3D cathode construction have the advantage of multi-component (support materials, co-catalysts, and catalyst promoting materials such as selected oxides) catalyst fabrication via electrospinning processes. Novel nanofiber materials and related fabrication approaches for the construction of 3D cathodes are still needed with proved catalytic performance and long-term stability.

### 7.2. Remaining Challenges and Perspectives

Since the technology of electrochemical reduction of CO_2_ is still nascent, novel fabrication technology and architecture design are needed for free-standing 3D cathode construction to enable breakthroughs in the development of active, selective, and stable electrocatalysts for CO_2_ reduction. In this regard, hierarchical porous structure engineering is promising, and a trend for both types of 3D cathode: free-standing and supported on a GDL. The importance of porous structures has been demonstrated in catalyst/electrode materials for CO_2_RR. By tailoring the pore size of porous materials from micropores to macropores, the performance of CO_2_RR can be improved. Macropores (>50 nm) provide facile transfer and diffusion of reactants and products in the reactions over the electrodes, while mesopores (2–50 nm) and micropores (<2 nm) can provide a high surface area to form more active sites with high dispersion. Thus, hierarchical porous materials with integrated properties are desirable for accelerating mass transport and improving CO_2_ conversion efficiency. Compared to conventional porous structures with uniform pore size distribution, hierarchical electrodes containing interconnected macro-, meso-, and micropores have demonstrated improved enhancement of material performance due to the increased specific surface area and mass transfer. Different strategies for the synthesis of hierarchical structures have been explored, such as self-assembly through hydrothermal or sol−gel reactions, hard porous template method with coating or sacrificing processes, and 3D printing [270]. As a new processing capability developed in recent years, 3D printing offers a novel approach for fabrication of nanoscale and hierarchical structures with desirable patterns [271,272]. Three-dimensional microarchitectured materials and devices using nanoparticle assembly by pointwise spatial printing has been developed recently [273]. This assembly method could be implemented by a variety of microdroplet generation methods for fast and large-scale fabrication of the hierarchical complex 3D materials. 3D porous electrodes have also been studied and used extensively for other applications as mentioned from the introduction of this review. Many lessons and success cases may have the potential to be transferred to technology for the electrochemical reduction of CO_2_, such as supported 3D composite structures based on GDL developed for fuel cells, and bioelectrochemical technologies [274,275].

Benefits of 3D compared to 2D cathodes were discussed based on the published literature. However, the performance of CO_2_ reduction is a function of a multitude of parameters, such as electrolyte concentration and composition, composition of catalysts or composite. It is difficult to compare performance from different studies without a relative standard criteria and protocol. While the majority of research to date has focused on performance evaluation of catalysts, there remain a lack of studies for large scale 3D cathodes and even fewer studies that combine 3D cathodes with optimized conditions for the best performance in a reactor system. It is clear that solely focusing on either catalysts, products, electrolytes, or cell design is unlikely to be effective. An optimization among all the components is ultimately required. Modelling studies for simulating the behavior of 3D cathodes and optimization at both component and system levels still needs to be conducted.

For both types of 3D structure, free-standing or supported on GDL, extension of the three phase boundaries inside the electrode is desired. In this case, both protonic and electronic conduction are required in the cathode, not only to realize low ohmic losses and a uniform current distribution, but also to extend the reaction sites of the three phases. Unfortunately, many 3D cathode studies only contain catalytic and electronic conduction components, without protonic conduction included. Low protonic and electronic conduction limit the catalytic active sites and lead to low current densities and high overpotential. This topic is beyond the scope of the 3D structure in this work and has been discussed and reviewed by others [276].

Fundamental understanding of structure–property relationships is a prerequisite for the development of highly efficient hierarchical porous structure electrocatalysts. Various in situ diagnosis tools should be used to directly investigate the reduction reactions and help identify optimal synthesis strategies for controlling the structure and components of catalysts. In addition, in situ characterization is important to help understand reaction dynamics and mechanisms for CO_2_ reduction with multi-products possible. Furthermore, the integrated information from the experimental results and diagnosis tools with multiphysics modeling may bring deeper understanding on the relationship of transport phenomena, fluid flow, and electrochemical reactions within the hierarchical porous structure electrode which would facilitate the rational design of high-performance catalysts as well as the prediction of the structure-performance relationship.

While further research and development will be made on 3D cathodes, cost of fabrication is another factor for consideration. Cost-effective, straightforward, and reproducible approaches for the fabrication of 3D cathodes should be persuaded for potential practical applications. The broad applications and fascinating properties of 3D porous electrodes will make them a highly active research area in the near future.

## Figures and Tables

**Table 1:** Selected products and equilibrium potentials of electrochemical reduction of CO_2_ [16] (this table is from an open access article).

Reaction	*E*^o^ (V vs. RHE)	Product
2H_2_O → O_2_ + 4H^+^ + 4e^−^	1.23	Oxygen Evolution Reaction (OER)
2H^+^ + 2e^−^ → H_2_	0	Hydrogen Evolution Reaction (HER)
*x*CO_2_ + nH^+^ + ne^−^ → product + *y*H_2_O	–	CO_2_ Reduction (CO_2_R)
CO_2_ + 2H^+^ + 2e^−^ → HCOOH_(aq)_	−0.12	Formic acid
CO_2_ + 2H^+^ + 2e^−^ → CO_(g)_ + H_2_O	−0.10	Carbon monoxide
CO_2_ + 6H^+^ + 6e^−^ → CH_3_OH_(aq)_ + H_2_O	0.03	Methanol (MeOH)
CO_2_ + 4H^+^ + 4e^−^ → C_(s)_ + 2H_2_O	0.21	Graphite
CO_2_ + 8H^+^ + 8e^−^ → CH_4(g)_ + 2H_2_O	0.17	Methane
2CO_2_ + 2H^+^ + 2e^−^ → (COOH)_2(s)_	−0.47	Oxalic acid
2CO_2_+ 8H^+^ + 8e^−^ → CH_3_COOH_(aq)_ + 2H_2_O	0.11	Acetic acid
2CO_2_ + 10H^+^ + 10e^−^ → CH_3_CHO_(aq)_ + 3H_2_O	0.06	Acetaldehyde
2CO_2_ + 12H^+^ + 12e^−^ → C_2_H_5_OH_(aq)_ + 3H_2_O	0.09	Ethanol (EtOH)
2CO_2_ 12H^+^ + 12e^−^ → C_2_H_4(g)_ + 4H_2_O	0.08	Ethylene
2CO_2_ + 14H^+^ + 14e^−^ → C_2_H_6(g)_ + 4H_2_O	0.14	Ethane
3CO_2_ 16H^+^ + 16e^−^ → C_2_H_5_CHO_(aq)_ + 5H_2_O	0.09	Propionaldehyde
3CO_2_ + 18H^+^ + 18e^−^ → C_3_H_7_OH_(aq)_ + 5H_2_O	0.10	Propanol (PrOH)
*x*CO + nH^+^ + ne^−^ → product + *y*H_2_O	*–*	CO Reduction (COR)
CO + 6H^+^ + 6e^−^ → CH_4(g)_ + H_2_O	0.26	Methane
2CO + 8H^+^ + 8e^−^ → CH_3_CH_2_OH_(aq)_ + H_2_O	0.19	Ethanol (EtOH)
2CO + 8H^+^ + 8e^−^ → C_2_H_4(g)_ + 2H_2_O	0.17	Ethylene

**Table 2:** Performance summary of 3D cathodes with different materials.

Supporting Materials		Catalysts	Fabrication Techniques	Cell and Performance	Ref.
**Self-supported**		Dense Cu nanowires on Cu mesh	Electrodeposition/oxidation/reduction	CO, 1 mA cm^−2^, 60% *FE*, −0.3 V	[116]
		Dense Cu nanowires on commercial Cu foam	Electrodeposition/oxidation/reduction	For specimen oxidized at 600 °C: 10 mA cm^−2^, CO/CHOO-, 80%, −0.5 V	[108]
		3D structured Au	PnP + electrodeposition	CO, 6.8 mA cm^−2^, 89% *FE*, −0.57 V	[104]
		Field’s metal-based	Chemical melting and sintering of metal nanoparticles	CHOO-, 12 mA cm^−2^, 80% *FE*, −0.8 V	[159]
		Cu foam (containing Cu_2_O)	Electrodeposition	CO + CHOO- + C_2_H_4_, 37.5 mA cm^−2^, (37% + 34% + 18% = 89%) *FE*, −1.1 V	[160]
		N doped porous carbon	Template-assisted chemical polymerization and carbonization	For 27 nm pore size: CO, 1.2 mA cm^−2^, 90% *FE*, −0.6 V, *FE* decays to 70% after 100 min	[134]
		N doped 3D graphene foam	CVD graphene deposition on Ni foam/doped with N by graphitic C3N4 at 800 °C	CO, 1.8 mA cm^−2^, 85% *FE*, −0.58 V, 5 h	[136]
		N doped carbon paper with atomically dispersed Ni	Carbonizing N-C precursor sprayed on Ni foil at 1000 °C	CO, 49 mA cm^−2^, 97% *FE*, −1.0 V	[161]
**1D Building Blocks**	PVDF nanofiber	Sb/Cu	Sn-decorated Cu-coated electrospun PVDF nanofiber	AEM assembly, CO, CD 104 mA cm^−2^, *FE* 82%, overpotential 0.9 V vs. RHE, 135 h	[164]
	MWNT/PyPBI	Au	Wet chemistry process with CNTs	1.0 M KCl, CO, CD 160 mA cm^−2^, *FE* 60%, overpotential 1.78 V vs. Ag/AgCl	[181]
	MWNT/PyPBI	Au	Wet chemistry process with CNTs	2.0 M KOH, CO, CD 158 mA cm^−2^, *FE* 49.4%, overpotential 0.94 V vs. SHE, 8 h	[122]
	MWNT on SS	SnO_x_	in situ or hydrothermal method	0.1 M KHCO_3_, formate, CD 5 mA cm^−2^, *FE* 64%, overpotential 1.4 V vs. SCE, 20 h	[187]
	MWNT on carbon paper	SnO_2_	Wet impregnation	0.5 M NaHCO_3_, formate, CD 80 mA cm^−2^, *FE* 27.2%, overpotential 1.7 V vs. SCE	[188]
	CNT Aerogel	Sn	Wet chemistry + calcination in H_2_	0.5 M KHCO_3_, formate, CD 24 mA cm^−2^, *FE* 82.7%, overpotential 0.96 V vs. RHE, 5.6 h	[191]
	CNT	HNCM	Wet chemistry + pyrolysis	0.1 M KHCO_3_, formate, CD 8 mA cm^−2^, *FE* 81%, overpotential 0.8 V vs. RHE, 36 h	[193]
	NCNT	Co_0.75_Ni_0.25_	Electrospinning + pyrolysis	0.5 M NaHCO_3_, CO, CD 13.4 mA cm^−2^, *FE* 85%, overpotential 0.9 V vs. RHE	[196]
	NCNT	Sn	Electrospinning + pyrolysis	0.5 KHCO_3_, formate, CD 11 mA cm^−2^, *FE* 62%, overpotential 0.69 V vs. RHE	[165]
	CC	SnO_2_	Hydrothermal reaction + calcination	0.5 M NaHCO_3_, formate, CD 45 mA cm^−2^, *FE* 87.2%, overpotential 0.88 V vs. Ag/AgCl	[200]
	ACF	Metal complexes	Wet chemistry	0.1 M KHCO_3_, CO, CD 70 mA cm^−2^, *FE* 70%, overpotential 1.5 V vs. SCE	[205]
	Cu_x_O NW	Sn-Cu_x_O	Anodizing, dehydration reduction, electroless deposition	0.1 M KHCO_3_, CO, CD 4.5 mA cm^−2^, *FE* 90%, overpotential 0.69 V vs. RHE	[207]
	CNF	Cu-CeO_x_	Electrospinning + pyrolysis	1.0 M KOH, CO, CD 100 mA cm^−2^, *FE* 59.2%, overpotential 0.60 V vs. RHE	[212]
	CNT	Cu-6.2%SnO_x_	Electrospinning + pyrolysis	0.1 M KHCO_3_, CO, CD 11.3 mA cm^−2^, *FE* 89%, overpotential 0.99 V vs. RHE	[213]
	CNT	Cu-30.2%SnOx	Electrospinning + pyrolysis	0.1 M KHCO_3_, formate, CD 4.0 mA cm^−2^, *FE* 77%, overpotential 0.99 V vs. RHE	[213]
**2D Building Blocks**	3D graphene	N doped 3D-GNE	CVD	*FE* 85% at −0.47 V, 5 h for CO_2_RR into CO	[136]
	3D graphene	Iron porphyrin/graphene hydrogel (FePGH	Hydrothermal with ascorbic acid	*FE* 96.2% at −0.39V and i = 0.42A/cm^2^, 19 h for CO_2_RR into CO	[227]
	NGA (N doped 3D graphene)	MnO	Hydrothermal	*FE* of 86% at −0.82 (RHE), 10 h for CO_2_RR into CO	[228]
	N-doped MEGO	Ni-N-MEGO	Microwave exfoliated 3D rGO	*FE* of 92% at –0.7V, 21 h for CO_2_RR into CO	[229]
	3D reduced GO	Pd0.5-In0.5/3D rGO	Chemical and hydrothermal	*FE* of 85.3% at −1.6V (Ag/AgCl) for CO_2_RR into CO	[106]
**3D Building Blocks**		Pt	Dual-templating approach	*FE* and yield for alcohol: 23.9% and 2.1 × 10^−8^ mol s^−1^ cm^−2^ at 51 mA cm^−2^, respectively. −2.05 V (vs. RHE) over 10 h	[241]
		Ag	Dealloy method	Overpotential <500 mV, *FE* 92% CO	[246]
	Cu Foam	SnOx@Sn-Cu/Sn core-shell	Electrodeposition Technique	406.7 +/− 14.4 mA cm^−2^ with C1 *FE* 98.0 +/− 0.9% at −0.70 V vs. RHE, 243.1 +/− 19.2 mA cm^−2^ with C1 *FE* 99.0 +/− 0.5% for 40 h at −0.55 V vs. RHE	[247]
	Carbon cloth	SnO_2_	Hydrothermal method	45 mA cm^−2^ at overpotential (−0.88 V), *FE* (87 +/− 2%), formate	[200]
	Graphene	SnO_2_	Hydrothermal method	10 mA cm^−2^, overpotential (−0.88 V) *FE* 93% for 18 h, formate	[197]
	CNT	RVC	CVD and EPD	200 mA cm^−2^ *FE* 100%, acetate	[105]
	CNT	N-doped carbon	MOF	Formate, −0.9 V vs. RHE, *FE* 81%	[193]
		F-doped carbon	Template method	*FE* 88.3% for CO at −1.0 V vs. RHE at 37.5 mA·cm^−2^ for 12 h	[251]
	PTFE	Cu	Sputtering method	7m KOH for 150 h −0.55 V vs. RHE, *FE* for ethylene 70%.	[96]
	N doped carbon	Cu/CuO	MOF	Formate *FE* 70.5% at −0.68 V. 30 h of 4.4 mA cm^−2^	[252]
	N-doped carbon	Ni	MOF	*FE*, CO over 71.9% at 10.48 mA cm^−2^ at overpotential −0.89 V. of 60 h	[253]
	CNT	Co	Electrospin	*FE* over 90% CO at 200 mA cm^−2^	[254]
